# LncRNA *BCCE4* Genetically Enhances the PD‐L1/PD‐1 Interaction in Smoking‐Related Bladder Cancer by Modulating miR‐328‐3p‐USP18 Signaling

**DOI:** 10.1002/advs.202303473

**Published:** 2023-09-13

**Authors:** Rui Zheng, Fang Gao, Zhenguang Mao, Yanping Xiao, Lin Yuan, Zhengkai Huang, Qiang Lv, Chao Qin, Mulong Du, Zhengdong Zhang, Meilin Wang

**Affiliations:** ^1^ Department of Environmental Genomics Jiangsu Key Laboratory of Cancer Biomarkers Prevention and Treatment Collaborative Innovation Center for Cancer Personalized Medicine Nanjing Medical University Nanjing 211166 China; ^2^ Department of Genetic Toxicology The Key Laboratory of Modern Toxicology of Ministry of Education Center for Global Health School of Public Health Nanjing Medical University Nanjing 211166 China; ^3^ Key Laboratory of Environmental Medicine Engineering Ministry of Education of China School of Public Health Southeast University Nanjing 210009 China; ^4^ Department of Urology Jiangsu Province Hospital of TCM Nanjing 210029 China; ^5^ Department of Integrated Traditional Chinese and Western Medicine Tumor Research Lab Nanjing 210028 China; ^6^ Department of Urology The First Affiliated Hospital of Nanjing Medical University Nanjing 210029 China; ^7^ Department of Biostatistics Center for Global Health School of Public Health Nanjing Medical University Nanjing 211166 China; ^8^ Institute of Clinical Research The Affiliated Taizhou People's Hospital of Nanjing Medical University Taizhou 225300 China; ^9^ The Affiliated Suzhou Hospital of Nanjing Medical University Suzhou Municipal Hospital Gusu School Nanjing Medical University Suzhou 215008 China

**Keywords:** bladder cancer, cigarette smoking, lncRNAs, PD‐L1/PD‐1 interaction, variants

## Abstract

Identification of cancer‐associated variants, especially those in functional regions of long noncoding RNAs (lncRNAs), has become an essential task in tumor etiology. However, the genetic function of lncRNA variants involved in bladder cancer susceptibility remains poorly understood. Herein, it is identified that the rs62483508 G > A variant in microRNA response elements (MREs) of lncRNA Bladder cancer Cell Cytoplasm‐Enriched abundant transcript 4 (*BCCE4*) is significantly associated with decreased bladder cancer risk (odds ratio = 0.84, *P* = 7.33 × 10^−8^) in the Chinese population (3603 cases and 4986 controls) but not in the European population. The protective genetic effect of the rs62483508 A allele is found in smokers or cigarette smoke‐related carcinogen 4‐aminobiphenyl (4‐ABP) exposure. Subsequent biological experiments reveal that the A allele of rs62483508 disrupts the binding affinity of miR‐328‐3p to facilitate USP18 from miRNA‐mediated degradation and thus specifically attenuates the downstream PD‐L1/PD‐1 interaction. LncRNA *BCCE4* is also enriched in exosomes from bladder cancer plasma, tissues, and cells. This comprehensive study clarifies the genetic mechanism of lncRNA *BCCE4* in bladder cancer susceptibility and its role in the regulation of the immune response in tumorigenesis. The findings provide a valuable predictor of bladder cancer risk that can facilitate diagnosis and prevention.

## Graphical abstract

Genetic variations in lncRNAs may lead to the gain or loss of miRNA‐binding elements that alter cancer susceptibility. Smoking‐related lncRNA *BCCE4* variant rs62483508 G > A can destroy a binding site for miR‐328‐3p and decrease *USP18* expression levels to attenuate the PD‐L1/PD‐1 interaction to enhance the antitumor immune response, ultimately resulting in the suppression of bladder tumorigenesis.

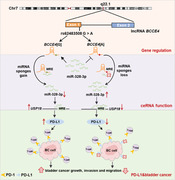

## 1 Introduction

Bladder cancer is the most common urological cancer globally,^[^ ^1^ ^]^ with ≈82 290 new diagnoses and 16 710 related deaths predicted in 2023 in the United States.^[^ ^2^ ^]^ In China, the incidence and mortality rates of bladder cancer have remained high in recent decades.^[^ ^3^ ^]^ Emerging evidence indicates that genetic factors and cigarette smoke exposure are vital factors in bladder cancer etiology.^[^ ^4^, ^5^, ^6^, ^7^ ^]^ Recently, genome‐wide association studies (GWASs) have become a powerful tool to interpret dozens of single nucleotide polymorphisms (SNPs) associated with multiple diseases,^[^ ^8^ ^]^ among these studies is our previous study on the first identification of a risk‐associated locus at 5q12.3 for bladder cancer in the Chinese population.^[^ ^9^ ^]^ However, only a few susceptibility loci are in protein‐coding regions, while the majority of such loci are in noncoding regions with unclear genetic function.^[^ ^10^, ^11^, ^12^ ^]^

Long noncoding RNAs (lncRNAs) comprise diverse and large lass of noncoding transcripts that are longer than 200 nucleotides.^[^ ^13^, ^14^, ^15^ ^]^ Accumulating studies have revealed that lncRNAs are emerging as key regulators of numerous cellular processes in gene expression patterns and epigenetic signatures,^[^ ^16^ ^]^ thereby mediating bladder cancer cell proliferation, migration, and invasion.^[^ ^17^, ^18^ ^]^ Many GWAS‐identified variants map to lncRNA regions, indicating that genetic variation in lncRNAs may play a vital role in the pathology of cancers.^[^ ^19^ ^]^ Mechanistically, lncRNAs are characterized as competing endogenous RNAs (ceRNAs), which can regulate mRNA levels by competitively binding to target genes harboring the same microRNA (miRNA) response elements (MREs).^[^ ^20^ ^]^ Moreover, susceptibility loci in lncRNAs may regulate cancer‐associated gene expression levels via the loss or gain of MREs to contribute to the risk of cancers,^[^ ^10^, ^21^ ^]^ but the effect of lncRNA variants on bladder cancer susceptibility has not been clarified. Accordingly, our present study aimed to identify bladder cancer‐causing variants in MREs in lncRNAs and help extend the understanding of the etiology of bladder cancer and reveal novel biomarkers implicated in bladder cancer.

In the present study, we initially constructed profiles of the MRE genetic variants in lncRNAs by high‐throughput RNA sequencing with subsequent evaluation and verification in a large‐scale genetic study. Cell and mouse assays were performed to systematically interpret the biological genetic effect of candidate causing variants in lncRNAs on bladder cancer susceptibility.

## 2 Results

### 2.1 Identification of a Cigarette Smoking‐Related Susceptibility Locus in MREs of lncRNA *BCCE4*

**Figure 1:**
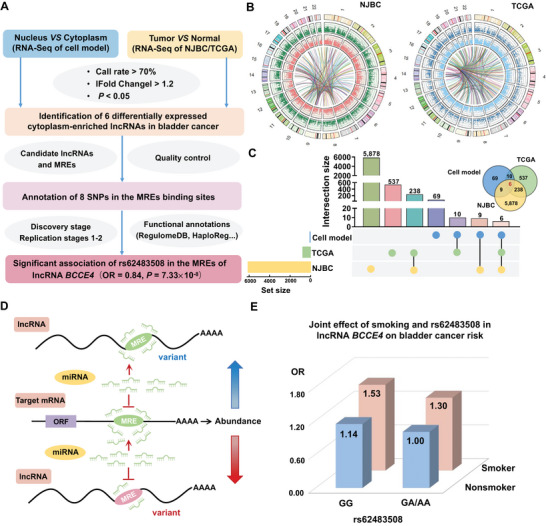
Procedures for identifying sponge‐acting lncRNAs and their MRE genetic variants associated with bladder cancer susceptibility. A) Schematic process of screening candidate lncRNAs and their MRE genetic variants. B) The Circos plot presents the genome‐wide view of 6 differentially expressed cytoplasm‐enriched lncRNAs and associated mRNAs in 29 pairs of bladder tumors and normal adjacent tissues from the NJBC datasets (left panel) and 19 pairs of bladder tumors and normal adjacent tissues from the TCGA database (right panel). C) Upset plots and Venn diagrams of the differential expression of lncRNAs in the cell model, NJBC dataset and TCGA database. D) Representation of the impact of MRE genetic variants in lncRNAs on miRNA‐mediated target gene expression. The blue represents variant in lncRNA gain a binding site for miRNA. The red represents variant in lncRNA loss a binding site for miRNA. E) Joint effect of smoking status and rs62483508 in lncRNA *BCCE4* on bladder cancer risk.

The flowchart of the identification strategy for new loci in MREs on miRNA‐ sponging lncRNAs is presented in **Figure** **1**A. We extracted cytoplasmic and nuclear and RNA from bladder cancer cell lines and used RNA sequencing analysis to specifically screen lncRNAs with ceRNA function (Figure S1A, Supporting Information). Moreover, we performed a transcriptomics‐guided screening based on lncRNA signatures of 29 pairs of bladder cancer tissues and corresponding normal tissues from the Nanjing Bladder Cancer (NJBC) dataset and 19 pairs of bladder cancer tissues and corresponding normal tissues from The Cancer Genome Atlas (TCGA) database to detect differentially expressed lncRNAs in bladder cancer (Figure S1B,C, Supporting Information). Based on the above results, we found 6 differentially expressed cytoplasm‐enriched lncRNAs in bladder cancer tissues (Figure 1B,C). To further filter the potential genetic variants that affect the sponge‐acting function of the above lncRNAs (Figure 1D), we used TargetScan and miRanda software and obtained 8 candidate SNPs in the MREs of *RP11‐132A1.4, KTN1‐AS1*, and *LINC00467* (Figure S2, Supporting Information). Next, we conducted a discovery study and the quality control, which was previously described,^[^ ^22^ ^]^ and found that only rs62483508 in exon 1 of *RP11‐132A1.4*, named as Bladder cancer Cell Cytoplasm‐Enriched abundant transcript 4 (*BCCE4)*, remained associated with a significantly decreased risk of bladder cancer (odds ratio (OR) = 0.81, 95% confidence interval (CI) = 0.70–0.94, *P*_adj_ = 4.47 × 10^−3^; Tables S1 and S2, Supporting Information). As shown in **Table** **1**; and Table S3 (Supporting Information), a similar effect of rs62483508 on bladder cancer risk was validated in two replication studies (OR = 0.85, 95% CI = 0.80–0.91, *P*_adj_ = 2.79×10^−6^). Moreover, combined analysis of the discovery dataset and two replication datasets showed that subjects with the rs62483508 A allele had a lower bladder cancer susceptibility than those with the G allele (OR = 0.84, 95% CI = 0.79–0.90, *P*_adj_ = 7.33×10^−8^). In addition, rs62483508 in lncRNA *BCCE4* appeared to may be functionally associated with bladder cancer susceptibility (Figure S3, Supporting Information).

**Table 1:** The association of rs62483508 in lncRNA *BCCE4* with the bladder cancer risk in the discovery, replication and combined stages.

Stage	Samples		Genotypes (GG/GA/AA)^a)^		MAF^b)^		OR (95% CI)^c)^	*P* ^c)^	*P*_het_ ^d)^	*I* ^2^
	Cases	Controls	Cases	Controls	Cases	Controls				
Discovery	580	1101	235/268/71	390/495/200	0.357	0.412	0.81 (0.70–0.94)	4.47 × 10^−3^		
Replication 1	1900	2676	722/929/249	940/1265/469	0.376	0.412	0.86 (0.79–0.94)	8.46 × 10^−4^		
Replication 2	1123	1209	425/518/174	409/523/258	0.388	0.437	0.83 (0.74–0.94)	2.11 × 10^−3^		
Replication	3023	3885	1147/1447/423	1349/1788/727	0.380	0.420	0.85 (0.80–0.91)	2.79 × 10^−6^	0.650	0.00%
Combined^e)^	3603	4986	1382/1715/494	1739/2283/927	0.376	0.418	0.84 (0.79–0.90)	7.33 × 10^−8^	0.554	0.00%

^a)^ Some individuals were not available for genotyping information;

^b)^ MAF, minor allele frequency, Minor allele frequency of the A allele;

^c)^ OR, odds ratio, CI, confidence interval, *P* for additive genetic model adjusted for age, sex, and smoking status in the logistic regression model;

^d)^ *P* value for heterogeneity;

^e)^ The discovery and two replication stages were combined by meta‐analysis.

We then performed stratified analyses and found that the effect of the rs62483508 G > A changed on bladder cancer risk was more obvious in older individuals (> 65 years), males and smokers in two replication stages (Figure S4, Supporting Information). Considering that smoking is the most important environmental factor for bladder cancer, we conducted joint analysis and revealed that smokers with the GG genotype exhibited a 1.53‐fold increased bladder cancer risk compared with nonsmokers with the GA/AA genotype (Figure 1E; and Table S4, Supporting Information), suggesting that the rs62483508 A allele may have a physiological relationship with cigarette smoke exposure. Furthermore, the association of rs62483508 with bladder cancer risk was not observed in individuals of European ancestry, which might be attributed to the different minor allele frequency (MAF) of rs62483508 in European populations versus Chinese populations (Table S5, Supporting Information). In addition, the results of power analysis showed that a sample size of 3603 cases and 4986 controls was sufficient (power = 97.4%) to determine a combined OR of 0.84 and α = 0.05 for rs62483508, with an MAF of 0.418 in controls (Figure S5, Supporting Information).

### 2.2 Expression Pattern of lncRNA *BCCE4* Across Bladder Cancer Tissues and Cells

**Figure 2:**
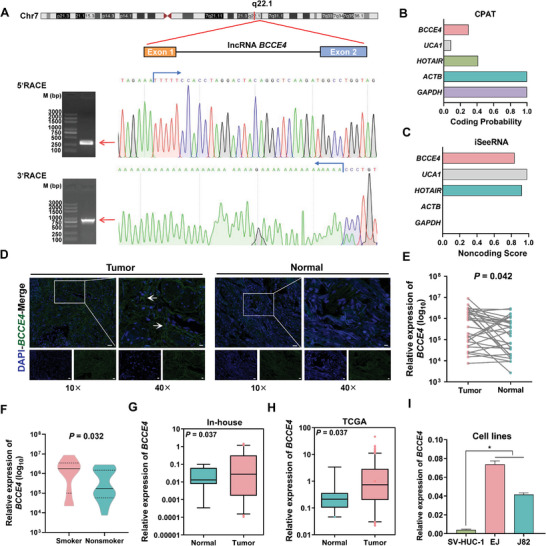
Characterization of lncRNA *BCCE4* in bladder cancer tissues and cells. A) Upper panel: Schematic representation of exons and transcripts of the lncRNA *BCCE4* transcript and its loci on human chromosome 7q22.1. Lower panel: RACE assays in EJ cells to identify the whole sequence of lncRNA *BCCE4*. B,C) The protein coding potential of lncRNA *BCCE4* was predicted by using CPAT and iSeeRNA. D) Representative fluorescence images of lncRNA *BCCE4* (green) in 30 pairs of bladder cancer tissues and normal adjacent tissues as detected by FISH analysis. Scale bars, 100 µm (10×) and 20 µm (40×). E) The relative IOD of lncRNA *BCCE4* as measured by FISH analysis. F) The relative IOD of lncRNA *BCCE4* as determined in smokers (*n* = 10) and nonsmokers (*n* = 20) by FISH analysis. G) The expression levels of lncRNA *BCCE4* as detected in bladder cancer tissues (*n* = 50) and normal tissues (*n* = 15) using RT‒qPCR. H) The expression of lncRNA *BCCE4* as quantified in bladder cancer tissues and normal tissues from the TCGA database. I) Differential expression of lncRNA *BCCE4* between normal cells (SV‐HUC‐1) and bladder cancer cells (EJ and J82) as detected by RT‒qPCR. Statistical significance was assessed using two‐tailed Student's *t*‐test. The values represent the mean ± SD. ^*^ *p* < 0.05.

By using 5′ and 3′ rapid amplification of cDNA ends (RACE) assays, we confirmed that the full‐length lncRNA *BCCE4* was 1141 bp in EJ cell lines, located on human chromosome 7q22.1 and had two exons (**Figure** **2**A; and Figure S6, Supporting Information). The results of the Coding Potential Assessment Tool (CPAT), iSeeRNA, and open reading frame (ORF) finder software revealed that lncRNA *BCCE4* had no protein‐coding potential (Figure 2B,C; and Figure S7, Supporting Information). To assess lncRNA *BCCE4* expression levels, we conducted fluorescence in situ hybridization (FISH) assays of 30 paired bladder cancer tissues and adjacent normal tissues and found that the expression of lncRNA *BCCE4* was significantly higher in bladder tumors than in normal tissues (Figure 2D,E), and its expression was also significantly higher in smokers than in nonsmokers (Figure 2F). We also evaluated the expression pattern of lncRNA *BCCE4* based on an in‐house dataset, TCGA database, and cell lines (SV‐HUC‐1, EJ, and J82), and the results showed increased lncRNA *BCCE4* expression levels in bladder cancer tissues, patients with high‐grade tumors and bladder cancer cells (Figure 2G–I; and Figure S8A, Supporting Information). Interestingly, the exoRBase database indicated that lncRNA *BCCE4* could be packaged into exosomes and enriched in blood. We found that lncRNA *BCCE4* was significantly higher in plasma exosomes acquired from bladder cancer patients than in those from cancer‐free control individuals (Figure S8B, Supporting Information). Additionally, lncRNA *BCCE4* expression was also upregulated in exosomes purified from EJ and J82 cell lines (Figure S8C, Supporting Information). Furthermore, TCGA and GTEx database analyses indicated that lncRNA *BCCE4* was widely expressed among multiple tissue types, revealing moderate abundance in bladder tumors or bladder tissues (Figure S9, Supporting Information). In addition, we aimed to assess the biological effect of lncRNA *BCCE4* on cellular phenotypes and found that lncRNA *BCCE4* knockdown significantly inhibited cell proliferation, colony formation, invasion and migration, and arrested the cell cycle at the G1 or S phase (Figure S10, Supporting Information).

### 2.3 Regulatory Effect of rs62483508 on lncRNA *BCCE4* Expression

**Figure 3:**
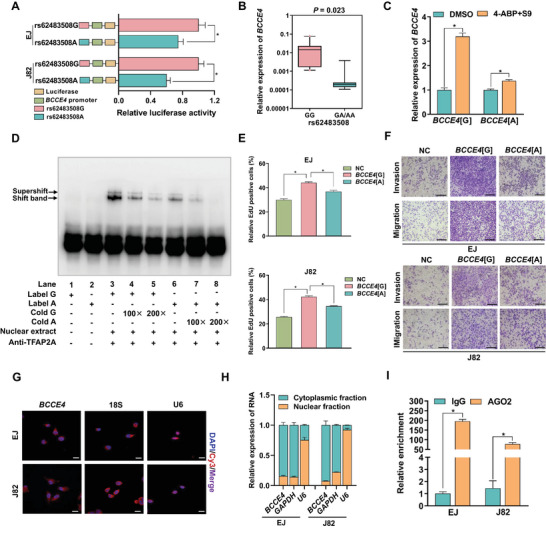
Effects of rs62483508 in the MREs of lncRNA *BCCE4* on bladder cancer cellular phenotypes. A) The allele‐specific effect of the constructs with the rs62483508 G or A allele in EJ and J82 cells by using dual luciferase reporter assays. B) An eQTL analysis of the correlation of lncRNA *BCCE4* expression levels and rs62483508 as measured in 29 bladder cancer tissues. C) The expression of lncRNA *BCCE4* in EJ and J82 cells transfected with *BCCE4* G or A vectors, namely, *BCCE4*[G] and *BCCE4*[A], and then exposed to 4‐ABP. D) TFAP2A supershift EMSAs with biotin‐labeled rs62483508 G or A probes in EJ cells. 100× and 200× represent 100‐fold and 200‐fold excess amounts of an unlabeled probe over that of the labeled probe. E) The NC, rs62483508 G, or A lentiviral vector was transfected into EJ and J82 cells, which were designated NC, *BCCE4*[G], and *BCCE4*[A], respectively. The effect of *BCCE4*[G] and *BCCE4*[A] overexpression on EJ (upper panel) and J82 (lower panel) cell proliferation as determined by EdU assay. F) The effect of *BCCE4*[G] and *BCCE4*[A] overexpression on the invasion and migration abilities of EJ (upper panel) and J82 (lower panel) cells as measured by a transwell assay. Scale bar, 20 µm. G) The cellular location of lncRNA *BCCE4* in EJ and J82 cells as detected by FISH analysis. Scale bar, 20 µm. H) The levels of lncRNA *BCCE4*, *GAPDH*, and *U6* in the subcellular fractions of EJ and J82 cells. I) RIP assays with an anti‐AGO2 antibody or IgG control performed on EJ and J82 cells. The expression levels of lncRNA *BCCE4* in the immunoprecipitates were measured by using RT‒qPCR. Statistical significance was assessed using two‐tailed Student's *t*‐test. The values represent the mean ± SD. ^*^ *p* < 0.05.

We predicted the function of the lncRNA *BCCE4* variant and found that the rs62483508 G > A variant could affect local folding structures (Figure S11, Supporting Information). Dual‐luciferase reporter assays revealed significantly lower relative luciferase activity in bladder cancer cell lines expressing the protective A allele construct than in those expressing the G allele construct (**Figure** **3**A). An expression quantitative trait locus (eQTL) analysis also showed that the A allele of rs62483508 markedly decreased lncRNA *BCCE4* expression levels in bladder cancer tissues (Figure 3B). Consistent with the results of joint analysis in smokers, we also found that the above eQTL effect was more obvious after exposing bladder cancer cell lines to the cigarette smoking carcinogen 4‐aminobiphenyl (4‐ABP, Figure 3C), indicating that the rs62483508 A allele might display a significant protective effect on bladder cancer risk in smokers. To investigate the mechanism by which rs62483508 might regulates the transcription of lncRNA *BCCE4*, we first performed transcription factor (TF) motif analysis by using the PROMO and JASPAR databases, and both predicted that TFAP2A might be a candidate TF (Figure S12A,B, Supporting Information). We next performed correlation analysis and found that *TFAP2A* was upregulated in bladder cancer tissues and positively associated with lncRNA *BCCE4* (Figure S12C–E, Supporting Information). Moreover, we conducted supershift electrophoretic mobility shift assays (EMSAs) and identified the preference of TFAP2A for the rs62483508 G allele probe (Figure 3D). These findings indicate that rs62483508 may regulate lncRNA *BCCE4* levels by altering the binding affinity for the transcription factor TFAP2A. We then assessed the genotype of rs62483508 in EJ and J82 cell lines by using Sanger sequencing, and found that EJ cells had the lncRNA *BCCE4* GG genotype and J82 cells had the lncRNA *BCCE4* GA genotype (Figure S13, Supporting Information). This led us to experimentally test the genetic effect of lncRNA *BCCE4* variants on the bladder cancer cellular phenotype using cells stably expressing the rs62483508 G (*BCCE4*[G]) or A (*BCCE4*[A]) allele after lentiviral vector transfection (Figure S14, Supporting Information). The results showed that *BCCE4*[A] dramatically suppressed the proliferation, migration, and invasion of bladder cancer cells compared with *BCCE4*[G] (Figure 3E,F; and Figure S15, Supporting Information). Consistent with the RNA‐Seq results based on different subcellular localizations, we performed a subcellular separation assay and FISH analysis in bladder cancer tissues and cell lines, and found that lncRNA *BCCE4* was mostly enriched in the cytoplasm (Figures 2D and 3G,H and; Figure S16, Supporting Information). The results of RNA immunoprecipitation (RIP) experiments also revealed that lncRNA *BCCE4* could directly bind to AGO2 (Figure 3I), which is a main member of the RNA‐induced silencing complex (RISC) and is related to mRNA repression regulated by miRNA. Interestingly, the expression of AGO2 was not significantly different after lncRNA *BCCE4* knockdown (Figure S17, Supporting Information). Taken together, these results indicate that this variant could affect the miRNA sponging function of the cytoplasmic lncRNA *BCCE4* involved in bladder cancer susceptibility.

### 2.4 LncRNA *BCCE4* Competitively Sponges miR‐328‐3p on USP18 in an Allele‐Specific Manner

**Figure 4:**
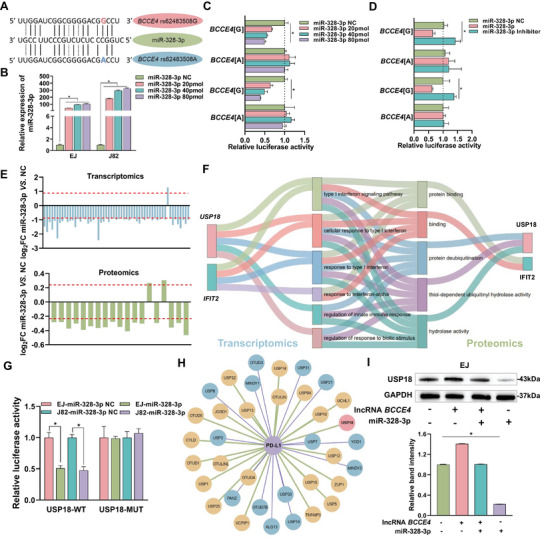
The lncRNA *BCCE4* variant regulates USP18 levels through a miR‐328‐3p‐dependent mechanism. The rs62483508 G or A lentiviral vector was transfected into EJ and J82 cells, namely, *BCCE4*[G] and *BCCE4*[A], respectively. A) Schematic diagram showing the MREs of lncRNA *BCCE4* containing the rs62483508 allele binding site for miR‐328‐3p. B) The expression of miR‐328‐3p in EJ and J82 cells transfected with different amounts of miR‐328‐3p mimic. C) Relative luciferase activity of the *BCCE4*[G] and *BCCE4*[A] constructs cotransfected with 20, 40, or 80 pmol of miR‐328‐3p mimic into EJ (upper panel) and J82 (lower panel) cells. D) Relative luciferase activity of the *BCCE4*[G] and *BCCE4*[A] constructs cotransfected with 80 pmol of miR‐328‐3p mimic or its inhibitor into EJ (upper panel) and J82 (lower panel) cells. E) Upper panel: Fold change (|FC| > 1.8, *P* < 0.05) of gene levels in EJ cells transfected with miR‐328‐3p mimic or control as detected by RNA sequencing. Lower panel: Fold change (|FC| >1.2, *P* < 0.05) of protein levels in EJ cells transfected with miR‐328‐3p mimic or control as detected by tandem mass tag (TMT). F) Representative Sankey diagram depicting the interaction between the overlapping differentially expressed genes/proteins and pathways. G) The relative luciferase activity as measured in EJ and J82 cells co‐transfected with miR‐328‐3p mimic or NC mimic and luciferase reporter vectors GP‐miRGLO‐USP18‐WT or GP‐miRGLO‐USP18‐MUT. H) The *PD‐L1* and USP family gene interaction network (*p* < 0.01) consists of *PD‐L1* (purple) and 36 USP family genes (blue, yellow, and pink). Yellow, significant positive correlations. Blue, significant negative correlations. Pink, target gene *USP18*. I) The protein levels of USP18 in EJ cells transfected with lncRNA *BCCE4* overexpression plasmids (lncRNA *BCCE4*) or miR‐328‐3p mimic (miR‐328‐3p) as detected by Western blotting. Statistical significance was assessed using two‐tailed Student's *t*‐test. The values represent the mean ± SD. ^*^ *p* < 0.05.

We used the RegRNA2.0 database and found that rs62483508 G > A lay within a putative binding site for miR‐328‐3p (**Figure** **4**A). Luciferase reporter assays revealed that in the presence of miR‐328‐3p, the *BCCE4*[G] group had markedly reduced relative luciferase activity compared with that of the *BCCE4*[A] group, and the inhibitory effect was dose‐dependent with respect to miR‐328‐3p concentration (Figure 4B,C; and Figure S18, Supporting Information). The above effect was indeed rescued when the miR‐328‐3p inhibitor was present (Figure 4D). We also assessed the effect of miR‐328‐3p on lncRNA *BCCE4* expression levels in EJ[G/G] and J82[G/A] cell lines, and revealed that miR‐328‐3p obviously reduced lncRNA *BCCE4* expression in *BCCE4*[G] allele (Figure S19, Supporting Information). To further prove the binding interaction between lncRNA *BCCE4* and miR‐328‐3p, we assessed the localization of lncRNA *BCCE4* and miR‐328‐3p and found that they were colocalized in the cytoplasm of bladder cancer cells (Figure S20A, Supporting Information). We then performed MS2‐RIP and found that MS2‐tagged lncRNA *BCCE4* plasmid enriched lots of miR‐328‐3p compared with mutant vectors and empty (Figure S20B, Supporting Information). Collectively, these findings indicate that lncRNA *BCCE4* might act as ceRNA regulated by sharing a common miR‐328‐3p binding site with targeted genes.

We then performed transcriptome and proteome expression profiling in EJ cell lines transfected with miR‐328‐3p mimic to identify the differentially expressed target genes or proteins of miR‐328‐3p (Figure 4E). The results of the Venn diagram showed that two predictors (USP18 and IFIT2) were both obviously expressed in both omics analyses (Figure S21, Supporting Information) and were enriched in the regulation of the innate immune response and protein deubiquitination (Figure 4F). Subsequently, we observed that *USP18* expression levels were markedly upregulated in bladder cancer tissues versus in normal tissues (Figures S22 and S23, Supporting Information). To confirm whether USP18 could be a target gene of miR‐328‐3p, we transfected a luciferase reporter vector harbouring the USP18 3′‐UTR into bladder cancer cell lines (Figure S24A, Supporting Information), and relative luciferase activity was then detected after miR‐328‐3p mimic transfection. As expected, we found that miR‐328‐3p overexpression significantly decreased luciferase activity in bladder cells transfected with USP18‐WT; however, we did not observe this effect when cells were transfected with USP18‐MUT (Figure 4G). Moreover, we found that miR‐328‐3p overexpression markedly reduced *USP18* levels, while the miR‐328‐3p inhibitor significantly upregulated *USP18* expression (Figure S24B, Supporting Information). We also found an obvious positive correlation between lncRNA *BCCE4* expression levels and *USP18* expression levels (Figure S24C, Supporting Information). We conducted RNA pull‐down assays and found that USP18 enriched in lncRNA *BCCE4* pull‐down products (Figure S24D, Supporting Information), indicating that lncRNA *BCCE4* may also regulate USP18 through other mechanisms. As described earlier, we revealed that miR‐328‐3p specifically binds to *BCCE4*[G] but not *BCCE4*[A]. Therefore, we transfected miR‐328‐3p mimic into bladder cancer cell lines stably overexpressing *BCCE4*[G] and revealed that miR‐328‐3p overexpression attenuated the oncogenic role of *BCCE4*[G] on bladder cancer proliferation, migration, and invasion (Figure S25, Supporting Information). Furthermore, we transfected USP18 overexpression plasmids into bladder cancer cell lines stably overexpressing *BCCE4*[A] and found that USP18 overexpression restored the tumor‐suppressing function of *BCCE4*[A] (Figure S26, Supporting Information). In addition, we used the CRISPR/Cas9 system to knock out the first exon region of lncRNA *BCCE4* and then transfected cells with rs62483508 G or A to generate independent *BCCE4*[G] or *BCCE4*[A] cell models. Based on these cell models, we also determined that bladder cancer cells expressing *BCCE4*[A] had lower USP18 or PD‐L1 levels and exhibited less malignant behavior than those expressing *BCCE4*[G] (Figure S27, Supporting Information). These observations imply that lncRNA *BCCE4* regulates bladder cancer cell malignant phenotypes by competitively sponging miR‐328‐3p on USP18 in an allele‐specific manner.

According to several studies, USP18 belongs to the USP subfamily of deubiquitinating enzymes (DUBs) that stabilize PD‐L1 expression levels and then influence the immune response.^[^ ^23^, ^24^ ^]^ This led us to assess the correlation coefficients between PD‐L1 and 65 DUBs; we found that *USP18* or lncRNA *BCCE4* was significantly correlated with *PD‐L1* in bladder cancer tissues (Figure 4H; and Figure S28A,B, Supporting Information). Indeed, we also found that miR‐328‐3p mimic transfection markedly decreased the protein levels of USP18 and that lncRNA *BCCE4* rescued the suppressive effect of miR‐328‐3p mimic on USP18 protein levels (Figure 4I; and Figure S28C, Supporting Information). Furthermore, we performed correlation analysis in bladder cancer tissues and found that lncRNA *BCCE4* levels exhibited a significant positive correlation with USP18 or PD‐L1 levels (Figure S28D–F, Supporting Information). Moreover, USP18 levels were also positively associated with PD‐L1 expression levels (Figure S28G, Supporting Information). Emerging evidence have reported that USP18 predominantly plays a role in removing interferon‐stimulated gene 15 (ISG15), regarded as a ubiquitin‐like protein (Ubl), from substrate proteins.^[^ ^25^, ^26^ ^]^ We first explored whether the protein stability of PD‐L1 was regulated by lncRNA *BCCE4* using a cycloheximide (CHX) chase assay and observed that endogenous PD‐L1 was unstable and degraded rapidly in bladder cancer cells transfected with si‐*BCCE4* (Figure S29A, Supporting Information). Furthermore, a recent study has reported that the E1‐like ubiquitin‐activating enzyme (UBE1L) relates with the interferon‐stimulated gene ISG15 that binds and inhibits substrate protein.^[^ ^27^ ^]^ As shown in Figure S29B (Supporting Information), we found that UBEL1 overexpression decreased the PD‐L1 protein expression. Subsequently, we employed Coimmunoprecipitation (Co‐IP) assays and found that USP18 could bind to ISG15 in EJ cells, as identified by an anti‐V5‐USP18 or anti‐HA‐ISG15 antibody (Figure S29C,D, Supporting Information). Moreover, we found that ISG15 conjugated with PD‐L1 in EJ cells (Figure S29E,F, Supporting Information). As expected, we also found that USP18 removed ISG15 from the PD‐L1 protein (Figure S29G,H, Supporting Information). Collectively, these findings suggest that lncRNA *BCCE4* augments USP18 levels to remove ISG15 from the PD‐L1 protein, thereby enhancing the stability of the target protein PD‐L1.

To further explore how lncRNA *BCCE4* affects T cells through the PD‐L1/PD‐1 interaction, we first incubated recombinant human PD‐1 Fc protein into EJ cells and found that the binding of PD‐1 on the bladder cancer cell surface increased as a result of lncRNA *BCCE4* overexpression, whereas it was significantly decreased while UPS18 knockdown (Figure S30A, Supporting Information). As expected, T‐cell cytotoxicity assays using activated Jurkat T cells also validated that lncRNA *BCCE4* significantly impaired the activity of cytotoxic T cells against bladder cancer cells (Figure S30B, Supporting Information). Moreover, we conducted another T‐cell cytotoxicity assay using EJ cells transfected with *BCCE4* over or si‐USP18 with activated human peripheral blood mononuclear cells (PBMCs) and found that EJ cells overexpressing lncRNA *BCCE4* responded poorly to incubation with activated human PBMCs (Figure S30C, Supporting Information). In addition, T cell cytotoxicity assays using activated Jurkat T cells or activated human PBMCs validated that bladder cancer overexpressing *BCCE4*[G] responded well to the treatment of PD‐L1 antibody (Figure S30D,E, Supporting Information). Taken together, these results provide evidence that USP18 inhibition may also decrease PD‐L1 expression to enhance the antitumor immune response.

### 2.5 Genetic Effect of lncRNA *BCCE4* on Bladder Tumors In Vivo

**Figure 5:**
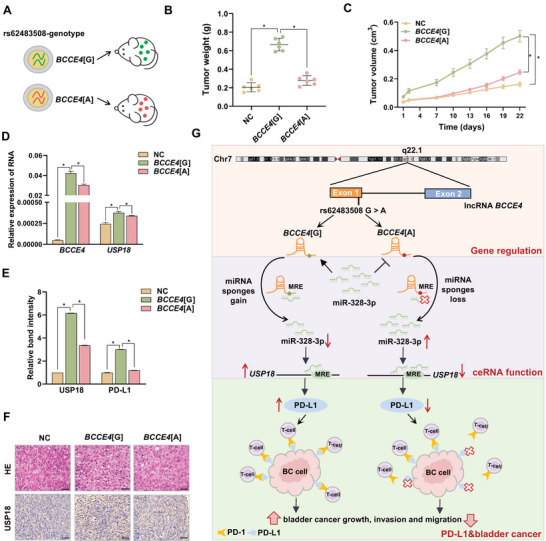
The effect of the lncRNA *BCCE4* variant in vivo. EJ cell lines transfected with the rs62483508 G, A or NC lentiviral vector were injected into humanized NCG mice, which were designated *BCCE4*[G], *BCCE4*[A], and NC, respectively. A) Schematic diagram representing the process of establishing the mouse model of the lncRNA *BCCE4* variant. The tumor weight (B) and tumor volumes (C) in the NC, *BCCE4*[G] and *BCCE4*[A] groups. D) The expression of lncRNA *BCCE4* and *USP18* in tumor tissues as determined by RT‒qPCR. E) The band intensity of USP18 and PD‐L1 in tumor tissues from NC, *BCCE4*[G], and *BCCE4*[A] groups as measured by Western blotting. F) Representative images of HE staining and IHC staining for USP18. Scale bar, 50 µm. G) Schematic diagram illustrating that G > A variation at rs62483508 in lncRNA *BCCE4* lost a miR‐328‐3p‐binding site and downregulated USP18 levels by missing its function as a miRNA sponge, which decreased PD‐L1 expression levels, inducing suppression of bladder cancer tumorigenesis. Statistical significance was assessed using two‐tailed Student's *t*‐test. The values represent the mean ± SD. ^*^ *p* < 0.05.

To unravel the potential role of the lncRNA *BCCE4* variant in bladder cancer growth in vivo, we subcutaneously injected EJ cells stably overexpressing *BCCE4*[G], *BCCE4*[A], or NC into six humanized NCG mice (**Figure** **5**A). Consistent with the in vitro results, both the weight and volume of tumors in the *BCCE4*[A] group were obviously reduced compared with those in the *BCCE4*[G] group (Figure 5B,C; and Figure S31A, Supporting Information). As shown in Figure 5D,E; and Figure S31B (Supporting Information), *BCCE4*[A] markedly attenuated lncRNA *BCCE4*, USP18, and PD‐L1 expression levels. Consistent with the results for the analysis of exosomes from bladder cancer cells and plasma, we found a higher level of tissue‐derived exosomal lncRNA *BCCE4* in the *BCCE4*[G] group than in the NC or *BCCE4*[A] group (Figure S31C, Supporting Information). Moreover, hematoxylin‐eosin (HE) and immunohistochemical (IHC) analyses revealed a weaker Ki67 index and lower USP18 and PD‐L1 expression levels in tumors derived from the *BCCE4*[A] group than in those derived from the *BCCE4*[G] group (Figure 5F; and Figure S31D–H, Supporting Information). Our data demonstrate that lncRNA *BCCE4* inhibits bladder cancer progression by acting as a ceRNA in an allele‐specific manner in vivo.

## 3 Discussion

To identify bladder cancer‐causing variants in MREs on noncoding regions, we first identified a susceptibility locus on MREs in lncRNA *BCCE4*, rs62483508 G > A, which decreases bladder cancer risk by combining with tobacco smoking. Mechanistically, the lncRNA *BCCE4* A allele loses a binding site for miR‐328‐3p, decreases USP18 expression levels by missing its function as a miRNA sponge, and thus downregulates PD‐L1 levels to restore the antitumour immune response in bladder cancer (Figure 5G).

In the cytoplasm, lncRNAs typically serve as molecular sponges of miRNAs to share MREs with the target mRNA 3′‐UTR, thereby regulating the expression levels of disease‐associated mRNAs targeted by miRNAs.^[^ ^28^ ^]^ Our group and other team have determined that the lncRNA‐miRNA‐mRNA crosstalk network could play an important role in bladder cancer risk and progression.^[^ ^21^, ^29^, ^30^ ^]^ Recently, emerging evidence has shown that GWAS‐identified loci in lncRNAs may influence the binding site activity of miRNAs involved in cancer susceptibility.^[^ ^10^, ^21^ ^]^ Accordingly, we interpreted RNA‐Seq transcriptome data of the cytoplasm and nucleus of bladder cancer cells and found that 6 cytoplasmic lncRNAs were significantly differentially expressed in bladder cancer tissues. Subsequently, we annotated MREs in the above lncRNAs and then conducted discovery and replication studies, which first revealed that rs62483508 G > A in the MREs of lncRNA *BCCE4* was significantly associated with decreased bladder cancer susceptibility in the Chinese populations. Intriguingly, this genetic effect was abolished in European population, suggesting that rs62483508 has specificity in Chinese individuals of different ethnicities.

Tobacco smoking is an essential risk factor for bladder cancer,^[^ ^22^, ^31^ ^]^ which could regulate the genetic effect of lncRNAs on diseases.^[^ ^32^ ^]^ However, the relationship between smoking behavior and bladder cancer‐associated lncRNA variants remains unclear. In this study, stratification analysis showed that the protective effect of rs62483508 on bladder cancer risk was present in smoking populations. Further joint analysis also identified that smokers with the rs62483508 GG genotype had a 53% higher risk of bladder cancer than nonsmokers with the rs62483508 GG/GA genotype. Interestingly, we treated *BCCE4*[G] or *BCCE4*[A] cell models with 4‐ABP, a critical tobacco smoke carcinogen in human bladder cancer,^[^ ^33^, ^34^ ^]^ and found that compared with *BCCE4*[A], *BCCE4*[G] could significantly upregulate lncRNA *BCCE4* expression levels after exposure to 4‐ABP. Moreover, lncRNA *BCCE4* was markedly upregulated in bladder cancer patients with a smoking history compared with nonsmokers. These findings indicate that rs62483508 A may act as a potential target for reducing bladder cancer susceptibility in smokers. In line with the findings observed from the populations, compared with *BCCE4*[G], *BCCE4*[A] substantially inhibited bladder cancer cell proliferation, migration and invasion in in vitro and in vivo studies, suggesting that *BCCE4*[A] ultimately contributes to inhibiting bladder cancer progression. Furthermore, the CRISPR/Cas9 system will be utilized to convert the genotype of lncRNA *BCCE4* rs62483508 in EJ and J82 cell lines, thereby directly confirming the effect of the rs62483508 genotype on the bladder cancer cell malignant phenotype.

The novel cytoplasmic lncRNA *BCCE4* with a length of 1141 bp was assessed via high‐throughput RNA sequencing and RACE analysis, and its expression was higher in bladder cancer tissues than in normal adjacent tissues according to FISH analysis. This pattern of expression was also observed in our in‐house samples, bladder cancer cell lines and TCGA database. Functionally, knockdown of lncRNA *BCCE4* significantly attenuated bladder cancer cell proliferation and clonogenicity by affecting the cell cycle. Interestingly, our previous studies have implied that exosomal noncoding RNA might serve as promising indicators in multiple diseases.^[^ ^30^, ^35^, ^36^ ^]^ In this study, analysis of the exoRBase database^[^ ^37^, ^38^ ^]^ and our data showed that lncRNA *BCCE4* was enriched in exosomes from bladder cancer cells, plasma and tissues. These findings indicate a tumor‐inducing role for lncRNA *BCCE4*.

Based on the above findings, we hypothesized that rs62483508 in lncRNA *BCCE4* might impact its target gene expression levels by destroying or gaining binding sites for miRNA. To validate this hypothesis, we first conducted RIP assays and found enrichment of lncRNA *BCCE4* with an anti‐AGO2 antibody, revealing that lncRNA *BCCE4* could directly interact with miRNAs. Subsequent in silico analysis, dual‐luciferase reporter assays, and correlation analysis indicated that the G > A transition at rs62483508 in lncRNA *BCCE4* might lose a binding site for miR‐328‐3p, resulting in the interaction of many miR‐328‐3p molecules with target genes. In addition, miR‐328‐3p has been reported to serve as a tumor suppressor by suppressing the malignant cell phenotype of bladder cancer.^[^ ^39^, ^40^ ^]^ Similarly, we also found that lncRNA *BCCE4* expression was negatively correlated with miR‐328‐3p expression in bladder cancer and revealed that miR‐328‐3p suppressed the oncogenic role of *BCCE4*[G]. To further investigate the target gene of miR‐328‐3p, we integrated transcriptomic and proteomic analyses and in vivo studies and found that *BCCE4*[A] significantly downregulated the expression of the target gene USP18 by destroying the miR‐328‐3p response element. Importantly, previous studies have reported that USP18 was upregulated in bladder cancer tissues and promoted the malignant cell phenotype of bladder cancer.^[^ ^41^, ^42^ ^]^ Consistent with the above findings, we also found that USP18 could restore the tumor‐suppressing function of *BCCE4*[A], indicating that USP18 plays an oncogenic role in bladder cancer. Although we have focused primarily on the mechanism of lncRNA *BCCE4* as a ceRNA, we will further explore the mechanism by which lncRNA *BCCE4* interacts with USP18. Ubiquitin‐specific peptidase 18 (USP18) is considered a critical multifunctional deubiquitinase that moderates the stability of key proteins to contribute to the carcinogenesis process.^[^ ^43^, ^44^ ^]^ Recently, PD‐1/PD‐L1‐targeting immune checkpoint inhibitors (ICIs) have been proven to be promising treatment modalities in bladder cancer.^[^ ^45^, ^46^ ^]^ Our study demonstrated that USP18 downregulation caused by *BCCE4*[A] could decrease the protein stability of PD‐L1, attenuate the PD‐L1/PD‐1 interaction and sensitize bladder cancer cells to T‐cell‐mediated killing. Moreover, bladder cancer cells with *BCCE4*[G] overexpression might exhibit a better response to anti‐PD‐L1 treatment in vitro; however, further validation based on a population study and in vivo models is necessary. Thus, our results suggest an effective way to inhibit bladder cancer progression in subjects carrying the rs62483508 G allele via PD‐L1/PD‐1 interaction.

In summary, this study highlighted a novel cytoplasm‐enriched lncRNA, *BCCE4*; we showed that the rs62483508 G > A variant of *BCCE4* lost a binding site for miR‐328‐3p, decreased USP18 levels, impaired the stability of PD‐L1, and then attenuated the PD‐L1/PD‐1 interaction, which significantly suppressed bladder cancer progression. Our findings not only shed new light on how noncoding variants contribute to a better understanding of bladder cancer pathogenesis, but also provide a new avenue for uncovering more novel biomarkers of bladder cancer in individuals with smoking history.

## 4 Experimental Section

### Study Populations and Genotyping

This study included a discovery stage and a two‐stage case‐control study of 3603 bladder cancer cases and 4986 cancer‐free controls and was performed to systematically assess the role of lncRNA variants in bladder cancer risk. The subjects in the discovery stage and first replication stage included 2480 cases and 3777 controls from Nanjing Medical University Affiliated Hospital. In the second stage, a total of 1123 cases and 1209 controls were recruited from Fudan University Shanghai Cancer Center. All cases were confirmed by pathologists, and cancer‐free controls were frequency‐matched to bladder cancer patients with age and sex. The subjects were from the Han Chinese population. Furthermore, the effects based on European populations from the database of Genotypes and Phenotypes (dbGaP, phs000346.v2.p2) were investigated.^[^ ^47^ ^]^ The UK Biobank (UKBB) resource used by this study were described in the previous study.^[^ ^48^ ^]^ Samples in the discovery stage were genotyped by using Illumina HumanOmniZhongHua chips, which were previously described in detail.^[^ ^9^ ^]^ LncRNA variants were genotyped by the TaqMan assay platform with the LightCycler LC480 (Roche, Switzerland). This study was approved by the Institutional Review Board of Nanjing Medical University (2016‐124).

### Transcriptomic and Proteomic Profiling

Based on total RNA extracted from cytoplasmic and nuclear fractions of bladder cancer cell lines, 29 paired bladder cancer tumors, and adjacent normal tissues obtained from the NJBC dataset or miR‐328‐3p mimic and control cells, whole‐transcriptome sequencing (RNA‐Seq) was conducted by Novogene Co., LTD. (Beijing, China) on the NovaSeq 6000 platform. Proteome profiling was performed in miR‐328‐3p mimic and control EJ cells by using tandem mass tag (TMT)‐based quantitative proteomic analysis (Novogene Co., Ltd., China).

### RACE Analyses and Coding Prediction of lncRNA *BCCE4*

5′ RACE and 3′ RACE analyses were performed to identify the transcriptional initiation and termination sites of lncRNA *BCCE4* with the GeneRacerTM Kit (Invitrogen, USA). The RACE assay gene‐specific PCR primer sequences are presented in the Table S6 (Supporting Information). The protein‐coding potential of lncRNA *BCCE4* was predicted by CPAT, iSeeRNA, and ORF finder.^[^ ^49^, ^50^ ^]^ *ACTB* and *GAPDH* served as coding RNA controls, while *UCA1* and *HOTAIR* served as noncoding RNA controls.

### FISH and Subcellular Separation

FISH with 5‐carboxyfluorescein (5‐FAM)‐labeled probe sequences specific for lncRNA *BCCE4* (GenePharma, China), anti‐USP18 (A16739, ABclonal, China), or anti‐PD‐L1 (GB11339A, ServiceBio, China) antibody was carried out to visualize the localization and measure the expression levels of lncRNA *BCCE4*, USP18, or PD‐L1 based on bladder cancer tissues and nontumor tissues following the manufacturer's instructions.^[^ ^36^ ^]^ Moreover, RNA FISH assays were performed using the Ribo FISH Kit (RiboBio Inc., China) to investigate the subcellular location of lncRNA *BCCE4* and the colocalization of lncRNA *BCCE4* and miR‐328‐3p in bladder cancer cells. In addition, nuclear and cytoplasmic RNA of EJ and J82 cells were prepared and isolated by using the Nuclear/Cytoplasmic Isolation kit (Thermo Fisher Scientific, USA). *GAPDH* and *U6* were regarded as the cytoplasmic and nuclear endogenous controls, respectively.

### Treatment of Bladder Cancer Cells with 4‐ABP

EJ and J82 cells were transiently transfected with *BCCE4*[G] or *BCCE4*[A] vectors. When the indicated cells reached nearly 70% confluence at the bottom of the petri dish, they were exposed to 0.5 mm 4‐ABP (Macklin, China) plus 6% rat live S9 (Biopredic, France) for 3 h.

### PD‐L1/PD‐1 Binding Measurement and T‐cell Cytotoxicity Assays

EJ cells transfected with lncRNA *BCCE4* overexpression plasmids or si‐USP18 were treated with 20 µg mL^−1^ recombinant human PD‐1 Fc protein (10377‐H02H, Sino Biological, China) at 25 °C for 2 h. The binding of PD‐1 on the bladder cancer cell surface was measured by fluorescence microscopy (ZEISS, Germany). Jurkat T cells or human PBMCs were activated via Dynabeads Human T‐Activator CD3/CD28 bead (11132D, Invitrogen, USA) treatment. The *BCCE4*[G]‐overexpressing or si‐USP18 EJ cells were then incubated with activated Jurkat T cells or activated PBMCs at different ratios for 24 h (activated PBMCs/ activated Jurkat T cells: bladder cancer cells). The cell counts were detected using a high‐content screening system and specific cytotoxicity was analyzed in a lactate dehydrogenase cytotoxicity assay (G1780, Promega, USA). The *BCCE4*[G]‐overexpressing EJ cells treated with anti‐PD‐L1 antibody (14‐5982‐82, eBioscience, USA) for 3 h were then co‐cultured with activated Jurkat T cells or activated PBMCs at different ratios for 24 h.

### Bladder Tumor Xenograft Model

Female NCG mice (4 weeks old, six mice per group) were randomly divided into the following three groups: NC, *BCCE4*[G] or *BCCE4*[A]. NC, *BCCE4*[G], or *BCCE4*[A] cells were harvested, and 1×10^7^ of the above cell lines were injected into the mice. The tumor volumes in each group were estimated from the length and width using the following formula: tumor volume (cm^3^) = (L×W^2^)/2. HE staining was performed to select representative areas, and IHC analysis was performed to identify the proliferation markers Ki67 (ab15580, Abcam, USA), USP18 (A16739, ABclonal, China), and PD‐L1 (#13 684, CST, USA). All animal results were approved by the Institutional Animal Care and Use Committee of Nanjing Medical University (IACUC‐2101023).

### Statistical Analysis

The distributions of characteristics between two groups were evaluated by Student's paired or independent *t*‐test and Pearson's *χ^2^*‐test. PS Power and Sample Size Calculations software (V.3.1; Nashville, TN) was applied to assess whether the sample size could effectively investigate the relationship between lncRNA *BCCE4* variants and the risk of bladder cancer.^[^ ^47^ ^]^ The OR and 95% CI for bladder cancer risk were obtained using multivariate logistic regression analysis to evaluate the genetic effect of lncRNA variants with adjustment for age, sex, and smoking status. The combined effect of lncRNA variants and smoking status was assessed by joint analysis. Spearman's correlation analysis was used to detect the relationship between lncRNA *BCCE4*, USP18, PD‐L1, or TFAP2A expression levels. All experiments were replicated at least three times. Quantitative data are shown as the means ± standard deviations (SD). A two‐sided *P* < 0.05 was considered to indicate a significant difference. Statistical analyses were conducted with R software (version 4.0.5) and PLINK (version 1.90). Additional experimental details are shown in the Materials and Methods of the Supporting Information.

## Conflict of Interest

The authors declare no conflict of interest.

## Author Contributions

R.Z. and F.G. contributed equally to this work. M.W., Z.Z., and M.D. designed and supervised the study. L.Y., Z.H., Q.L., and C.Q. contributed to study subject recruitment and biological sample collection. R.Z., F.G., and Y.X. contributed to the functional experiments. R.Z., F.G., and Z.M. performed the statistical analyses and summarized results. R.Z. and F.G. prepared the manuscript.

## Supporting information

Supporting Information

## Data Availability

The data that support the findings of this study are available from the corresponding author upon reasonable request.
