# Supplementary material for: LncRNA BCCE4 Genetically Enhances the PD‐L1/PD‐1 Interaction in Smoking‐Related Bladder Cancer by Modulating miR‐328‐3p‐USP18 Signaling
Source: Adv Sci (Weinh). 2023 Sep 13;10(30):2303473. doi: 10.1002/advs.202303473 (PMC10602555; doi:10.1002/advs.202303473)

ADVANCED SCIENCE
Open Access

# Supporting Information

for *Adv. Sci.*, DOI 10.1002/advs.202303473

LncRNA *BCCE4* Genetically Enhances the PD-L1/PD-1 Interaction in Smoking-Related Bladder Cancer by Modulating miR-328-3p-USP18 Signaling

*Rui Zheng, Fang Gao, Zhenguang Mao, Yanping Xiao, Lin Yuan, Zhengkai Huang, Qiang Lv, Chao Qin, Mulong Du, Zhengdong Zhang and Meilin Wang**

ADVANCED SCIENCE
Open Access

Supporting Information

for *Adv. Sci.*, DOI 10.1002/advs.202303473

# LncRNA *BCCE4* Genetically Enhances the PD-L1/PD-1 Interaction in Smoking-Related Bladder Cancer by Modulating miR-328-3p-USP18 Signaling

*Rui Zheng, Fang Gao, Zhenguang Mao, Yanping Xiao, Lin Yuan, Zhengkai Huang, Qiang Lv, Chao Qin, Mulong Du, Zhengdong Zhang and Meilin Wang**

**Supplementary Materials and Methods**

**Supplementary Tables**

**Table S1.** Characteristics of the individuals included in the discovery and replication stages.

**Table S2.** The association between 8 candidate variants in MREs and the risk of bladder cancer in the discovery stage.

**Table S3.** The association of rs62483508 in lncRNA *BCCE4* with bladder cancer risk in different genetic models.

**Table S4.** Joint effect of rs62483508 in lncRNA *BCCE4* and smoking status on bladder cancer risk.

**Table S5.** The association of rs62483508 in lncRNA *BCCE4* with the bladder cancer risk in European populations.

**Table S6.** Sequences of primers and probes used in the study.

**Supplementary Figures**

**Figure S1.** Profiling of sponge-acting lncRNA by RNA-Seq.

**Figure S2.** Overview of 8 candidate variants in MREs of lncRNAs.

**Figure S3.** Functional annotations of 8 candidate variants in MREs of lncRNAs.

**Figure S4.** Forest plot of the stratified analysis of the association between rs62483508 and bladder cancer risk in two replication stages.

**Figure S5.** Power calculations for the sample size.

**Figure S6.** Characterization of full-length lncRNA *BCCE4* in EJ cell lines by RACE assays.

**Figure S7.** Characterization of the protein-coding potential for lncRNA *BCCE4*.

**Figure S8.** Expression of lncRNA *BCCE4* in bladder cancer.

**Figure S9.** Expression of lncRNA *BCCE4* in pan-tissues.

**Figure S10.** The effect of lncRNA *BCCE4* knockdown on bladder cancer cellular phenotypes.

**Figure S11.** Prediction of the effects of rs62483508 G > A change on lncRNA *BCCE4* folding structure.

**Figure S12.** Prediction of lncRNA *BCCE4* transcription factors.

**Figure S13.** The genotype of lncRNA *BCCE4* rs62483508 in EJ and J82 cell lines.

**Figure S14.** Determination of the transfection efficiency of *BCCE4*[G] or *BCCE4*[A] stable overexpression.

**Figure S15.** Biological function of lncRNA *BCCE4* rs62483508 G > A in bladder cancer cellular phenotypes.

**Figure S16.** The cellular localization of lncRNA *BCCE4* in bladder cancer cells.

**Figure S17.** Expression of AGO2 in bladder cancer cells transfected with si-*BCCE4*.

**Figure S18.** Function relevance of the lncRNA *BCCE4* variant.

**Figure S19.** The effect of miR-328-3p on lncRNA *BCCE4* expression levels in an allele-specific manner.

**Figure S20.** The interaction of between lncRNA *BCCE4* and miR-328-3p.

**Figure S21.** Genome-wide gene expression variation after miR-328-3p modulation.

**Figure S22.** *USP18* and *IFIT2* expression patterns in bladder cancer tissues.

**Figure S23.** The expression of USP18 in pan-tissues and bladder cancer tissues.

**Figure S24.** Identification of the target gene of miR-328-3p.

**Figure S25.** The effect of miR-328-3p regulation by lncRNA *BCCE4* rs62483508 G > A on bladder cancer cellular phenotype.

**Figure S26.** The effect of USP18 regulation by lncRNA *BCCE4* rs62483508 G > A on bladder cancer cellular phenotype.

**Figure S27.** Biological function of lncRNA *BCCE4* rs62483508 G > A in lncRNA *BCCE4* knockout bladder cancer cell lines.

**Figure S28.** The correlation between lncRNA *BCCE4*, USP18 and PD-L1 in bladder cancer.

**Figure S29.** Effect of USP18 on PD-L1 stability.

**Figure S30.** Effect of lncRNA *BCCE4* on T-cell cytotoxicity via the PD-L1/PD-1 interaction.

**Figure S31.** The expression of lncRNA *BCCE4*, USP18 and PD-L1 in vivo.

## Supplementary Materials and Methods

### NJBC dataset and public clinical databases

A total of 29 patients providing paired bladder cancer tissues and normal adjacent tissues were recruited from the Nanjing Bladder Cancer (NJBC) dataset. Briefly, the NJBC study followed inclusion criteria including a recent diagnosis of bladder cancer by histopathology and clinical examination. Cancer-free controls were selected from the same geographical region during the same period. Moreover, The Cancer Genome Atlas (TCGA), the Genotype-Tissue Expression (GTEx), The Gene Expression Omnibus (GEO) databases (GSE13507, GSE65635 and GSE7476), the Human Protein Atlas (HPA) and the Functional Annotation of the Mammalian Genome project (FANTOM5) database were used to evaluate the expression patterns of candidate genes.

### Cell lines and cell culture

Human bladder cancer (EJ and J82) and normal cells (SV-HUC-1) were obtained from the Cell Bank of Type Culture Collection of the Chinese Academy of Science, Shanghai, China. EJ cells were maintained in RPMI-1640 medium (Biological Industries, USA), SV-HUC-1 cells were maintained in F12K medium (Biological Industries, USA), and J82 cells were cultured in MEM (KeyGen Biotech, China), containing 10% heat-inactivated foetal bovine serum (FBS) (Biological Industries, USA), 100 μg/ml streptomycin (Gibco, USA) and 100 U/ml penicillin (Gibco, USA) in a humidified incubator containing 5% $CO_2$ at 37 °C.

### Total RNA and exosomal RNA isolation

Total RNA from cell lines and bladder cancer or normal tissues was extracted with TRIzol reagent (Invitrogen, USA). To extract exosomes from tissues, a small piece of

tissue was weighed, briefly sliced on dry ice and then incubated in the dissociation mixture for 10-15 min at 37°C. The suspension was passed through 70 μm filters, and the filtrate was supplemented with a protease and phosphatase inhibitor cocktail. The exosome lysates were clarified by differential centrifugation and filtered with 0.22 μm filters. The supernatant was centrifuged at 150,000 × g for 2 h at 4 ℃, and the precipitate was resuspended in cold PBS with a protease and phosphatase inhibitor cocktail. Then, the exosomes were purified by SEC (size-exclusion chromatograph) using Exosupur® columns (Echobiotech, China), and the fractions were concentrated to 200 μL by 100 kDa molecular weight cut-off Amicon® Ultra spin filters (Merck, Germany). RNA from exosomes was isolated by using a RNeasy Mini Kit (Qiagen, Germany). The technical assistance provided by EchoBiotech Co. Ltd. (Beijing, China). Exosomal RNA was isolated from cell culture medium, plasma or above tumor tissue extract products with the exoRNeasy Serum/Plasma Maxi kit (Qiagen, Germany) according to the manufacturer's instructions. The quality and quantity of extracted RNA were measured using a Nanodrop 2000 spectrophotometer (Thermo Fisher Scientific, USA).

**Reverse transcription and quantitative real-time PCR (RT–qPCR)**

The cDNA templates were synthesized from lncRNA and mRNA using a high-capacity cDNA reverse transcription kit (Invitrogen, USA), while the cDNA templates were synthesized from miRNA by using Reverse Transcriptase XL (AMV) (TaKaRa, Japan), Recombination RNase Inhibitor (TaKaRa, Japan) and dNTP Mixture (TaKaRa, Japan). RT–qPCR for candidate gene and miRNA expression levels was performed with the SYBR Green RT–PCR Kit (Vazyme Biotech, China) and a Roche Light

Cycler 480 II system (Roche, Switzerland). Glyceraldehyde-3-phosphate dehydrogenase (*GAPDH*) or *U6* served as an internal control. The RT–qPCR primer sequences are presented in **Table S6**.

**Vectors, lentivirus construction and transfection**

To obtain the lentiviral vector expressing lncRNA *BCCE4*, full-length lncRNA *BCCE4* cDNA containing the rs62483508 G or rs62483508 A allele was synthesized (GeneChem, China) using the CV032 infection system (GeneChem, China), which was confirmed by Sanger sequencing. Small interfering RNAs (siRNAs) were constructed to target lncRNA *BCCE4* or USP18 (GenePharma, China), whereas lncRNA *BCCE4*, USP18 or UBE1L overexpression plasmids were constructed by Genebay Biotech Co., Ltd. (China). In addition, the miR-328-3p mimic or inhibitor was synthesized and purified by RiboBio Inc. (China). The bladder cancer cell lines were transiently transfected with the above plasmids by using Lipofectamine 3000 (Invitrogen, USA) following the manufacturer’s protocol.

**Luciferase reporter assays**

The lncRNA *BCCE4* encompassing the corresponding rs62483508 G or A allele was inserted into the GPL3 luciferase constructs (Genebay, China). The whole sequence of lncRNA *BCCE4* or the 3′ UTR of USP18 sequences containing the complementary sequence for the miR-328-3p seed region was cloned into the 3′ end of the GP-miRGLO luciferase vector (Genechem, China). The EJ and J82 cell lines with miRNA mimic or inhibitor cotransfected with 60 ng pRL-SV40 Renilla vectors or 0.5 μg reporter vectors were seeded in 24-well plates using Lipofectamine 3000 reagent. The next day, the luciferase activity was detected by a Dual-Luciferase Assay System (Promega, USA). The relative luminescent signal was normalized to the Renilla

signals.

**Cell proliferation and colony formation assays**

The indicated EJ and J82 cells were seeded in 96-well plates and incubated at 37°C for 6 h, 24 h, 48 h or 72 h. The proliferation of the indicated bladder cancer cells was measured by a Cell Counting Kit-8 (CCK-8) (Dojindo, Japan) assay in accordance with the manufacturer's protocols. Absorbance was obtained at 450 nm (OD450) with a microplate reader (Infinite M200 Pro, Switzerland). Cell proliferation activity was tested by using a Cell-Light EdU DNA cell proliferation kit (RiboBio, China). For the colony formation assay, the indicated cells were plated onto 6-well culture plates until visible colonies appeared, and the culture medium was replaced every 3 days. The colonies were fixed in 95% methanol for 20 min, stained with 0.1% crystal violet (Beyotime Institute of Biotechnology, China) for 20 min and counted by ImageJ software.

**Cell invasion and migration assays**

To investigate the effect of lncRNA *BCCE4*, *BCCE4* variants, miR-328-3p or USP18 on bladder cancer cell lines, Matrigel (Corning, UAS) was coated with the upper chamber of 8-μm transwell chambers (Corning, UAS). After 24 h of culture, the indicated cells were seeded into the upper chamber with 100 μl of serum-free medium, and culture medium containing 10% FBS was added to the bottom chamber. After incubation for 24 h, the indicated cells on the upper chamber were removed, fixed with 95% methanol for 20 min and stained with 0.1% crystal violet for 20 min. Images were captured under five microscopic fields. Migration assays were also conducted with Transwell inserts without Matrigel. The migrated cells were detected and quantified according to the processes described for the invasion assay.

**Flow cytometry analysis of the cell cycle**

The indicated cells were harvested and fixed in 70% ethanol at -20°C for at least 18 h, and then stained with 500 µl propidium iodide (PI) solution for 15 min. The stained cells were analyzed by flow cytometry (BD Biosciences, USA).

**Electrophoretic mobility shift assays (EMSAs)**

Nuclear extracts of EJ cells were incubated with synthetic double-stranded and 3' biotin-labeled oligonucleotides corresponding to the rs62483508 sequence by using a LightShift Chemiluminescent EMSA Kit (Thermo Scientific, USA) according to the manufacturer's instructions. For the competitive binding assay, unlabeled probes at 100-fold or 200-fold excess oligonucleotides were added to the binding reaction 20 min prior to the addition of the labeled probes. Supershift EMSA was conducted to explore specific DNA–protein products by using a TFAP2A antibody (ab108311, Abcam, USA). The EMSA probe sequences are listed in **Table S6**.

**Western blotting**

The indicated cell lysates were obtained by using RIPA lysis buffer mixed with 0.5% phenylmethylsulfonyl fluoride (PMSF), and then centrifuged at 12,000 rpm for 15 min. The concentration of protein was determined by a bicinchoninic acid (BCA) protein assay (Beyotime Institute of Biotechnology, China). Equal amounts of proteins were resolved by sodium dodecyl polyacrylamide gel electrophoresis (SDS–PAGE) and transferred onto polyvinylidene fluoride (PVDF) membranes (Millipore, USA). After blocking, membranes were incubated overnight at 4°C with anti-AGO2(#03-110, Millipore, USA), anti-USP18 (ab168478, Abcam, USA), anti-PD-L1 (#13684, CST, USA), and anti-GAPDH (#5174, CST, USA). GAPDH served as a loading control for normalization. The immune complexes were examined by

using a Bio-Rad gel imaging system (Tanon, China).

**RIP assay**

The RIP assay was conducted with a Magna RIP RNA-Binding Protein Immunoprecipitation kit (Millipore Magna, USA) according to the manufacturer's instructions. In brief, lysates of EJ and J82 cell lines were incubated with RIP buffer containing IgG controls or AGO2 antibody (#03-110, Millipore, USA)-conjugated magnetic beads. The coprecipitated RNAs were detected by RT–qPCR.

**MS2-RIP assay**

Cells were co-transfected with pcDNA3.1-MS2-*BCCE4*-WT, pcDNA3.1-MS2-*BCCE4*-MUT respectively and pcDNA3.1-MS2-GST (HRP-66001, Proteintech, China). The RIP assay was conducted on the cells as above described after 24 h and the expression of miR-328-3p was measured by RT–qPCR.

**RNA pull-down assay**

The RNA pull-down assay was conducted with the Pierce™ Magnetic RNA–Protein Pull-Down kit (Thermo Fisher Scientific) following the manufacturer's instructions. Briefly, 5 μg labeled RNA was mixed with $1 \times 10^7$ *BCCE4*[G] or *BCCE4*[A] cell extracts and incubated for 1 h, followed by incubation with Dynabeads M-280 Streptavidin (Invitrogen) at 4 °C overnight. Subsequently, the pull-down complexes were eluted by denaturation in 1× protein loading buffer for 5 min, and were detected by Western blotting.

**Cycloheximide chase assay**

EJ cells were transfected with si-*BCCE4* for 24 h, and the cell medium of the above cells was then treated with 20 μmol/L cycloheximide (CHX) at the indicated time points. Afterwards, the cells were collected, and Western blotting was performed to

determine USP18 or PD-L1 protein expression at different time points.

**Coimmunoprecipitation (Co-IP)**

EJ cells were lysed in IP lysis buffer (P0013, Beyotime, China) containing a 1× protease inhibitor cocktail (P1005, Beyotime, China) and 1mM PMSF (ST506, Beyotime, China). Lysates were then clarified by centrifugation at 13,000 × g for 30 min at 4°C. Immunoblotting was performed with antibodies against the epitopes HA-ISG15 (IP: #3274, CST, USA; IB: #2367, CST, USA), V5-USP18 (IP: R960-25, Invitrogen, USA; IB: #13202, CST, USA), and Flag-PD-L1 (IP: #8146, CST, USA; IB: #2386, CST, USA); the blots were then incubated with the HRP-conjugated goat anti-mouse or anti-rabbit antibody, and the bands were detected by using a Bio-Rad gel imaging system (Tanon, China).

**CRISPR/Cas9-mediated knockout of lncRNA *BCCE4***

LncRNA *BCCE4*-deficient EJ cell lines (*BCCE4*-KO)-were designed and synthesized by using CRISPR/Cas9 technology (GenScript Biotech, China). Single guide RNAs (sgRNAs) targeting lncRNA *BCCE4* were cloned into the LentiCRISPR V2 vector. The sgRNA sequence targeting lncRNA *BCCE4* sites are shown in **Table S6**. The knockout of lncRNA *BCCE4* was validated through Sanger sequencing and RT–qPCR.

**URLs**

CPAT: http://lilab.research.bcm.edu/

iSeeRNA: https://sunlab.cpy.cuhk.edu.hk/iSeeRNA/webserver.html

ORF finder: https://www.ncbi.nlm.nih.gov/orffinder/

RNAfold Webserver: http://rna.tbi.univie.ac.at/cgi-bin/RNAWebSuite/RNAfold.cgi

RegulomeDB: https://www.regulomedb.org/regulome-search/

HaploReg: http://pubs.broadinstitute.org/mammals/haploreg/haploreg_v4.php

3DSNP: https://omic.tech/3dsnpv2/

TCGA: https://portal.gdc.cancer.gov

GEO: https://www.ncbi.nlm.nih.gov/geo/

GTEx: https://gtexportal.org/home/

HPA dataset: https://www.proteinatlas.org/

PROMO: https://alggen.lsi.upc.es/cgibin/promo_v3/promo/promoinit.cgi?dirDB=

TF_8.3

JASPAR: https://jaspar.genereg.net/

R: https://www.r-project.org/

## Supplementary Tables

**Table S1.** Characteristics of the individuals included in the discovery and replication stages.

| Variables | Discovery | | Replication 1 | | Replication 2 | | Combined | |
|---|---|---|---|---|---|---|---|---|
| | **Cases (n = 580)** | **Controls (n = 1,101)** | **Cases (n = 1,900)** | **Controls (n = 2,676)** | **Cases (n = 1,123)** | **Controls (n = 1,209)** | **Cases (n = 3,603)** | **Controls (n = 4,986)** |
| Age (Mean ± SD) | 64.7 ± 12.1 | 64.5 ± 12.1 | 64.8 ± 12.6 | 64.5 ± 10.2 | 61.1 ± 12.7 | 60.6 ± 11.0 | 63.6 ± 12.7 | 63.5 ± 11.0 |
| Sex (%) | | | | | | | | |
| Male | 481 (82.9%) | 905 (82.2%) | 1,520 (80.0%) | 1,923 (71.9%) | 905 (80.6%) | 924 (76.4%) | 2,906 (80.7%) | 3,752 (75.3%) |
| Female | 99 (17.1%) | 196 (17.8%) | 380 (20.0%) | 753 (28.1%) | 218 (19.4%) | 285 (23.6%) | 697 (19.3%) | 1,234 (24.7%) |
| Smoking Status (%) | | | | | | | | |
| Never | 313 (54.0%) | 717 (65.1%) | 1,009 (53.1%) | 1,748 (65.3%) | 678 (60.4%) | 722 (59.7%) | 2,000 (55.5%) | 3,187 (63.9%) |
| Ever | 264 (45.5%) | 384 (34.9%) | 891 (46.9%) | 921 (34.4%) | 427 (38.0%) | 487 (40.3%) | 1,582 (43.9%) | 1,792 (36.0%) |
| Missing | 3 (0.5%) | 0 (0.0%) | 0 (0.0%) | 7 (0.3%) | 18 (1.6%) | 0 (0.0%) | 21 (0.6%) | 7 (0.1%) |

**Table S2.** The association between 8 candidate variants in MREs and the risk of bladder cancer in the discovery stage.

| LncRNA | SNP | Position[a] | Allele | MAF | | OR (95% CI) | $P_{crude}$ | OR (95% CI)[b] | $P_{adj}$[b] |
|---|---|---|---|---|---|---|---|---|---|
| | | | | Cases | Controls | | | | |
| *BCCE4* | rs62483508 | Chr7: 100951802 | G>A | 0.357 | 0.412 | 0.80 (0.69-0.92) | $2.36 \times 10^{-3}$ | 0.81 (0.70-0.94) | $4.47 \times 10^{-3}$ |
| | rs28608158 | Chr7: 100951712 | C>A | 0.056 | 0.051 | 1.11 (0.81-1.52) | 0.524 | 1.13 (0.82-1.56) | 0.458 |
| | rs10273309 | Chr7: 100954922 | A>T | 0.083 | 0.081 | 1.03 (0.79-1.34) | 0.814 | 1.02 (0.78-1.33) | 0.886 |
| *KTN1-AS1* | rs11847588 | Chr14: 56025646 | G>A | 0.197 | 0.206 | 0.94 (0.79-1.13) | 0.531 | 0.95 (0.79-1.14) | 0.586 |
| | rs755073 | Chr14: 56025783 | T>C | 0.463 | 0.489 | 0.90 (0.78-1.04) | 0.163 | 0.90 (0.77-1.04) | 0.137 |
| | rs77127093 | Chr14: 56026136 | T>C | 0.080 | 0.094 | 0.83 (0.64-1.08) | 0.164 | 0.83 (0.64-1.08) | 0.164 |
| | rs2209229 | Chr14: 56026384 | C>G | 0.150 | 0.155 | 0.96 (0.79-1.18) | 0.707 | 0.94 (0.77-1.16) | 0.578 |
| *LINC00467* | rs1055565 | Chr1: 211605697 | G>A | 0.337 | 0.330 | 1.03 (0.89-1.20) | 0.682 | 1.04 (0.89-1.21) | 0.649 |

Abbreviations: Chr, chromosome; MAF, minor allele frequency; OR, odds ratio; CI, confidence interval.

[a] Based on the NCBI database, build 37.

[b] *P* for additive genetic model adjusted for age, sex and smoking status in the logistic regression model.

**Table S3.** The association of rs62483508 in lncRNA *BCCE4* with bladder cancer risk in different genetic models.

| SNP | Stage | Genotypes | Cases | | Controls | | OR (95% CI)[a] | $P^{a}$ |
|---|---|---|---|---|---|---|---|---|
| | | | N | % | N | % | | |
| rs62483508 | Discovery | GG | 235 | 40.9 | 390 | 35.9 | 1.00 (ref.) | |
| | | GA | 268 | 46.7 | 495 | 45.7 | 0.91 (0.73-1.13) | 0.389 |
| | | AA | 71 | 12.4 | 200 | 18.4 | 0.61 (0.44-0.83) | $2.01 \times 10^{-3}$ |
| | | Dominant model | | | | | 0.82 (0.67-1.01) | $6.52 \times 10^{-2}$ |
| | | Recessive model | | | | | 0.64 (0.48-0.86) | $2.85 \times 10^{-3}$ |
| | Replication 1 | GG | 722 | 38.0 | 940 | 35.2 | 1.00 (ref.) | |
| | | GA | 929 | 48.9 | 1,265 | 47.3 | 0.96 (0.84-1.09) | 0.508 |
| | | AA | 249 | 13.1 | 469 | 17.5 | 0.70 (0.58-0.84) | $1.52 \times 10^{-4}$ |
| | | Dominant model | | | | | 0.89 (0.79-1.01) | $5.94 \times 10^{-2}$ |
| | | Recessive model | | | | | 0.72 (0.61-0.85) | $1.20 \times 10^{-4}$ |
| | Replication 2 | GG | 425 | 38.0 | 409 | 34.4 | 1.00 (ref.) | |
| | | GA | 518 | 46.4 | 523 | 43.9 | 0.96 (0.80-1.16) | 0.684 |
| | | AA | 174 | 15.6 | 258 | 21.7 | 0.66 (0.52-0.84) | $6.12 \times 10^{-4}$ |
| | | Dominant model | | | | | 0.86 (0.73-1.02) | $9.23 \times 10^{-2}$ |
| | | Recessive model | | | | | 0.67 (0.54-0.84) | $3.21 \times 10^{-4}$ |
| | Replication | GG | 1,147 | 38.1 | 1,349 | 34.9 | 1.00 (ref.) | |
| | | GA | 1,447 | 47.9 | 1,788 | 46.3 | 0.96 (0.86-1.06) | 0.401 |
| | | AA | 423 | 14.0 | 727 | 18.8 | 0.68 (0.59-0.78) | $1.33 \times 10^{-7}$ |
| | | Dominant model | | | | | 0.88 (0.79-0.97) | $8.59 \times 10^{-3}$ |
| | | Recessive model | | | | | 0.70 (0.61-0.79) | $6.31 \times 10^{-8}$ |

| | | | | | | | |
|---|---|---|---|---|---|---|---|
| Combined | GG | 1,382 | 38.5 | 1,739 | 35.1 | 1.00 (ref.) | |
| | GA | 1,715 | 47.8 | 2,283 | 46.1 | 0.95 (0.86-1.04) | 0.280 |
| | AA | 494 | 13.7 | 927 | 18.8 | 0.67 (0.59-0.76) | $1.67 \times 10^{-9}$ |
| | Dominant model | | | | | 0.87 (0.79-0.95) | $1.92 \times 10^{-3}$ |
| | Recessive model | | | | | 0.69 (0.61-0.78) | $9.55 \times 10^{-10}$ |

[a] *P* for additive genetic model adjusted for age, sex and smoking status in the logistic regression model.

**Table S4.** Joint effect of rs62483508 in lncRNA *BCCE4* and smoking status on bladder cancer risk.

| SNP | Stage | Smoking status | Genotypes | Cases/Controls | OR (95% CI)[a] | $P^a$ |
|---|---|---|---|---|---|---|
| rs62483508 | Replication | smokers | GG | 498/478 | 1.44 (1.23-1.68) | $3.41 \times 10^{-6}$ |
| | | nonsmokers | GG | 640/868 | 1.12 (0.99-1.28) | $7.72 \times 10^{-2}$ |
| | | smokers | GA/AA | 818/921 | 1.23 (1.08-1.40) | $1.91 \times 10^{-3}$ |
| | | nonsmokers | GA/AA | 1,043/1,590 | 1.00 (ref.) | |
| | | $P_{interaction}$ (multiplicative) | | | | 0.112 |
| | | $P_{trend}$ | | | | $4.26 \times 10^{-5}$ |
| | Combined | smokers | GG | 608/619 | 1.53 (1.34-1.76) | $1.09 \times 10^{-9}$ |
| | | nonsmokers | GG | 763/1,117 | 1.14 (1.01-1.28) | $3.25 \times 10^{-2}$ |
| | | smokers | GA/AA | 970/1,162 | 1.30 (1.16-1.46) | $9.17 \times 10^{-6}$ |
| | | nonsmokers | GA/AA | 1,229/2,044 | 1.00 (ref.) | |
| | | $P_{interaction}$ (multiplicative) | | | | 0.814 |
| | | $P_{trend}$ | | | | $1.68 \times 10^{-7}$ |

Abbreviations: OR, odds ratio; CI, confidence interval.

[a] Adjusted for age and sex in the logistic regression model.

**Table S5.** The association of rs62483508 in lncRNA *BCCE4* with the bladder cancer risk in European populations.

| SNP | Chr | Position[a] | Major | Minor | Database | MAF | | OR (95% CI)[b] | *P*[b] |
|---|---|---|---|---|---|---|---|---|---|
| | | | | | | Cases | Controls | | |
| rs62483508 | 7 | 100951802 | G | A | dbGaP | 0.174 | 0.179 | 0.97 (0.91-1.04) | 0.422 |
| | | | | | UKBB | 0.189 | 0.178 | 1.07 (0.96-1.19) | 0.224 |

Abbreviations: Chr, chromosome; MAF, minor allele frequency; OR, odds ratio; CI, confidence interval; dbGap, the database of Genotypes and Phenotypes; UKBB, UK Biobank.

[a] Based on the NCBI database, build 37.

[b] Adjusted for age and sex in the logistic regression model.

**Table S6.** Sequences of primers and probes used in the study.

| Experiments | Description | Sequences | |
|---|---|---|---|
| **TaqMan** | **SNP ID** | | |
| | rs62483508 | Primer | F: AGGAGCGGAGCAGAGGCA<br>R: AACTGCATAGCCCCGGG |
| | | Probe | FAM-CGCGGGAGCTGCAGGCCGT<br>HEX-CGCGCGGGAGCTGCAGGTCG |
| **RACE** | **Gene name** | | |
| | lncRNA *BCCE4* | 5’RACE | CCTTCCATTGAGATCTGCACATCCTGTGT |
| | | 3’RACE | GGGACGCCTCCAAAGGAACCCAGTAA |
| **FISH** | **Gene name** | | |
| | lncRNA *BCCE4* | 5-FAM-labeled *BCCE4*_probe1 | CGT+TTCCCGTGTCCT+TCCATTGAGAT |
| | | 5-FAM-labeled *BCCE4*_probe2 | T+TTGGAGGCGTCCCACT+TCCTTGCT |
| | | 5-FAM-labeled *BCCE4*_probe3 | AGTGAT+TTCT+TCAGT+TCCACAT+TTG |
| **RT–qPCR** | **Gene name** | | |
| | lncRNA *BCCE4* | Forward | CACCTCTCCTCTGACGGGAA |
| | | Reverse | CGCGTTCTCTTAGCGGATCT |
| | miR-328-3p | Forward | ACACTCCAGCTGGGCTGGCCCTCTCTGC |
| | | Reverse | TGGTGTCGTGGAGTCG |
| | | RT | CTCAACTGGTGTCGTGGAGTCGGCAATTCAGTTGAGACGG AAGG |
| | *U6* | Forward | CGCTTCGGCAGCACATATACTAAAATTGGAAC |
| | | Reverse | GCTTCACGAATTTGCGTGTCATCCTTGC |
| | | RT | AAAATATGGAACGCTTCACG |
| | *USP18* | Forward | CCTGAGGCAAATCTGTCAGTC |
| | | Reverse | CGAACACCTGAATCAAGGAGTTA |
| | *GAPDH* | Forward | GGAGCGAGATCCCTCCAAAAT |
| | | Reverse | GGCTGTTGTCATACTTCTCATGG |
| **EMSA** | rs62483508-EM-FAP | Biotin-CGGCGGGGACGACCTGCAGCTCC | |
| | rs62483508-EM-RAP | GGAGCTGCAGGTCGTCCCCGCCG-Biotin | |
| | rs62483508-EM-FGP | Biotin-CGGCGGGGACGGCCTGCAGCTCC | |

| | | | |
|---|---|---|---|
| | rs62483508-EM-RGP | GGAGCTGCAGGCCGTCCCCGCCG-Biotin | |
| | rs62483508-EM-FAJ | CGGCGGGGACGACCTGCAGCTCC | |
| | rs62483508-EM-RAJ | GGAGCTGCAGGTCGTCCCCGCCG | |
| | rs62483508-EM-FGJ | CGGCGGGGACGGCCTGCAGCTCC | |
| | rs62483508-EM-RGJ | GGAGCTGCAGGCCGTCCCCGCCG | |
| **CRISPR/Cas9** | **sgRNA** | | |
| | sgRNA-1 | GGCACGCACCUCUCCUCUGA | |
| | sgRNA-2 | GGGAUUCAGCCGAGAAGCCA | |
| | sgRNA-3 | GCGGAGAAAUUGGAUCGGCG | |
| **siRNA/mimic** | **Gene name** | | |
| | *BCCE4*-siRNA | Sense | GAAGCAAUUUACACAGGAUTT |
| | | Anti-sense | AUCCUGUGUAAAUUGCUUCTT |
| | USP18-siRNA | Sense | GCCCUUGUUUGUCCAACAUTT |
| | | Anti-sense | AUGUUGGACAAACAAGGGCTT |
| | miR-328-3p mimic | Sense | CUGGCCCUCUCUGCCCUUCCGU |
| | | Anti-sense | ACGGAAGGGCAGAGAGGGCCAG |

## Figure legends

**Figure S1. Profiling of sponge-acting lncRNA by RNA-Seq. (A)** Representative heatmap of differentially expressed lncRNAs in the cytoplasm and nucleus of bladder cancer cells (EJ and J82). EJ_N and J82_N represent the nucleus of EJ and J82 cells, respectively. EJ_C and J82_C represent the cytoplasm of EJ and J82 cells, respectively. **(B)** The differentially expressed lncRNAs between 29 pairs of bladder tumors and normal adjacent tissues from NJBC dataset are listed in the volcano plot. **(C)** The differentially expressed lncRNAs between 19 pairs of bladder tumors and normal adjacent tissues from TCGA database are listed in the volcano plot.

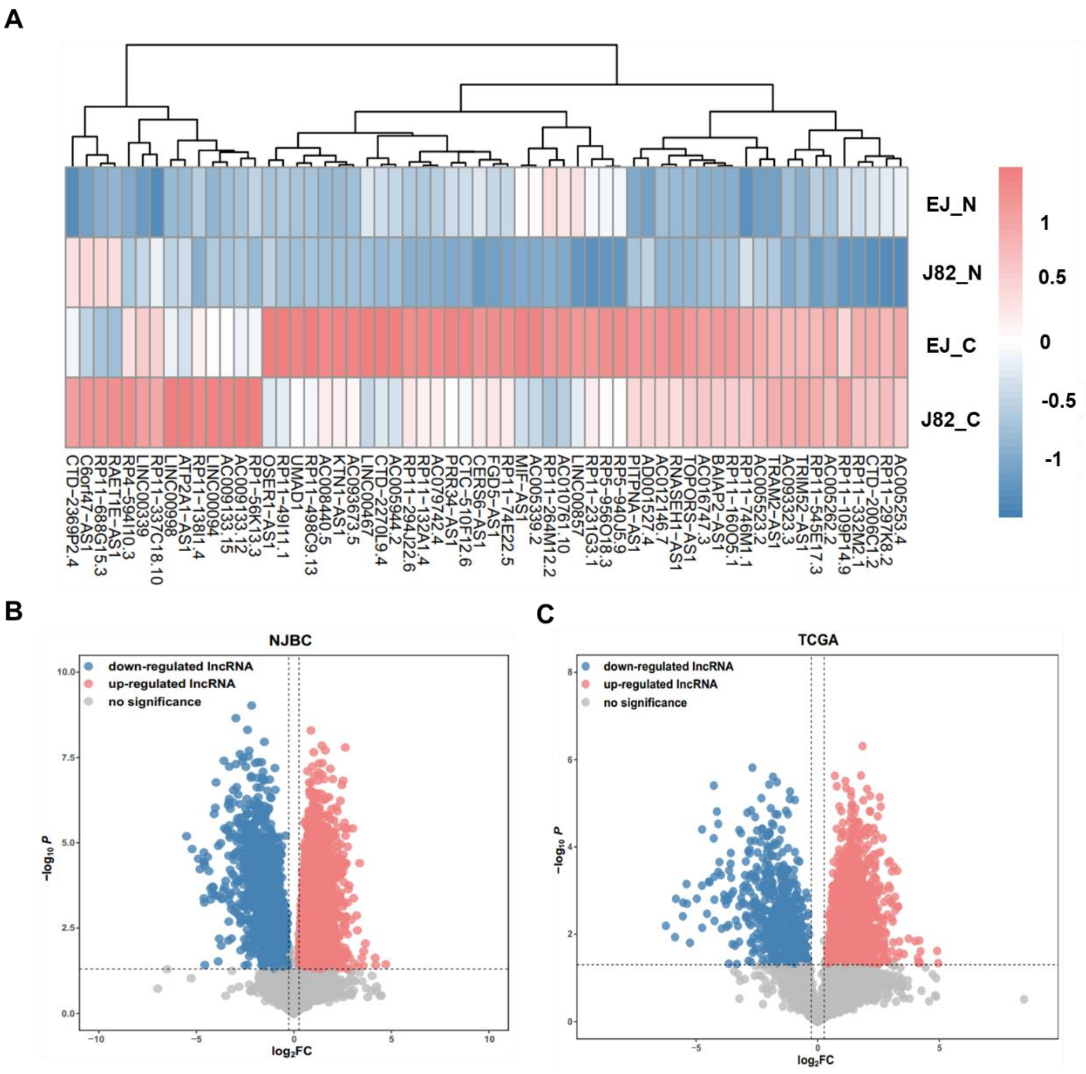

**Figure S2. Overview of 8 candidate variants in MREs of lncRNAs.** Circos plot showing 8 candidate variants in MREs of lncRNAs in bladder cancer.

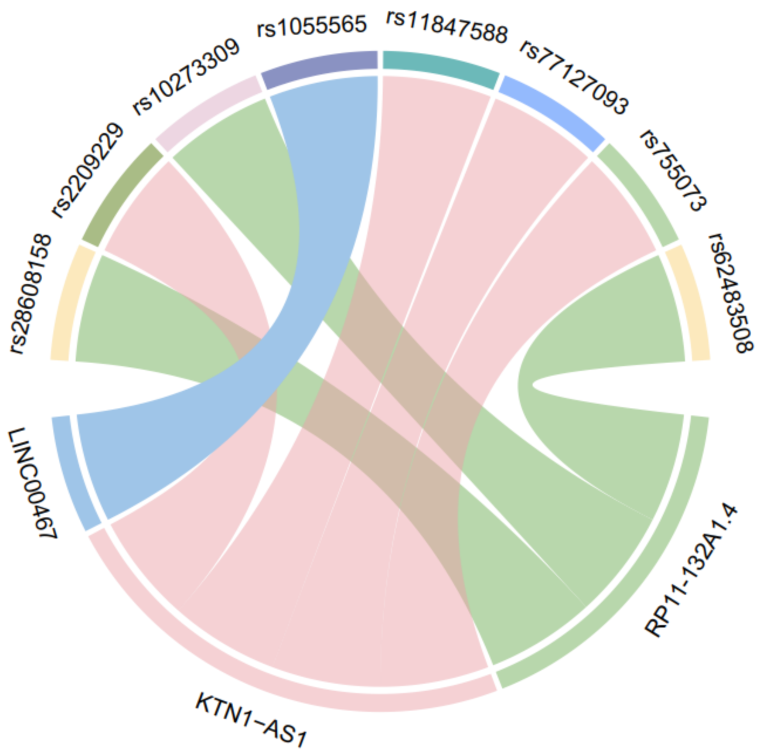

**Figure S3. Functional annotations of 8 candidate variants in MREs of lncRNAs.**

Functional annotations with scores from RegulomeDB, HaploReg and 3DSNP for the 8 candidate variants in MREs of lncRNAs.

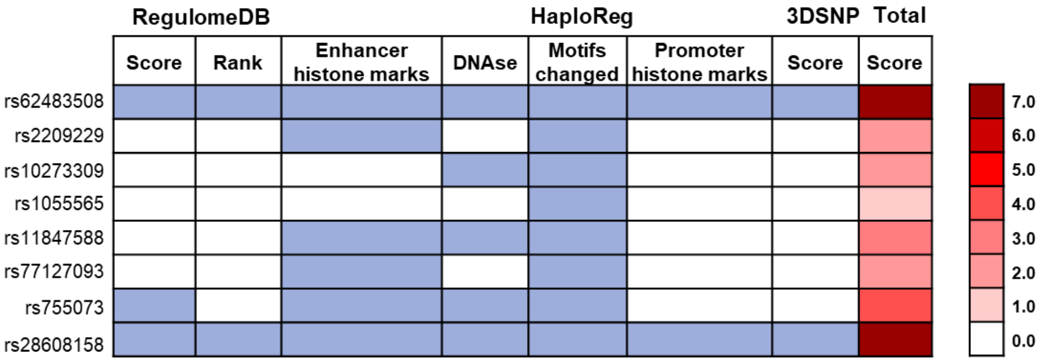

**Figure S4. Forest plot of the stratified analysis of the association between rs62483508 and bladder cancer risk in two replication stages.** The risk effect of the two replication stages was assessed in subgroups stratified by age (≤ 65 or > 65), sex (male or female) and smoking status (never or ever). OR was estimated with the adjustment of sex, age and smoking status when appropriate. OR, odds ratio; CI, confidence interval; $P_{het}$, $P$ value for the heterogeneity.

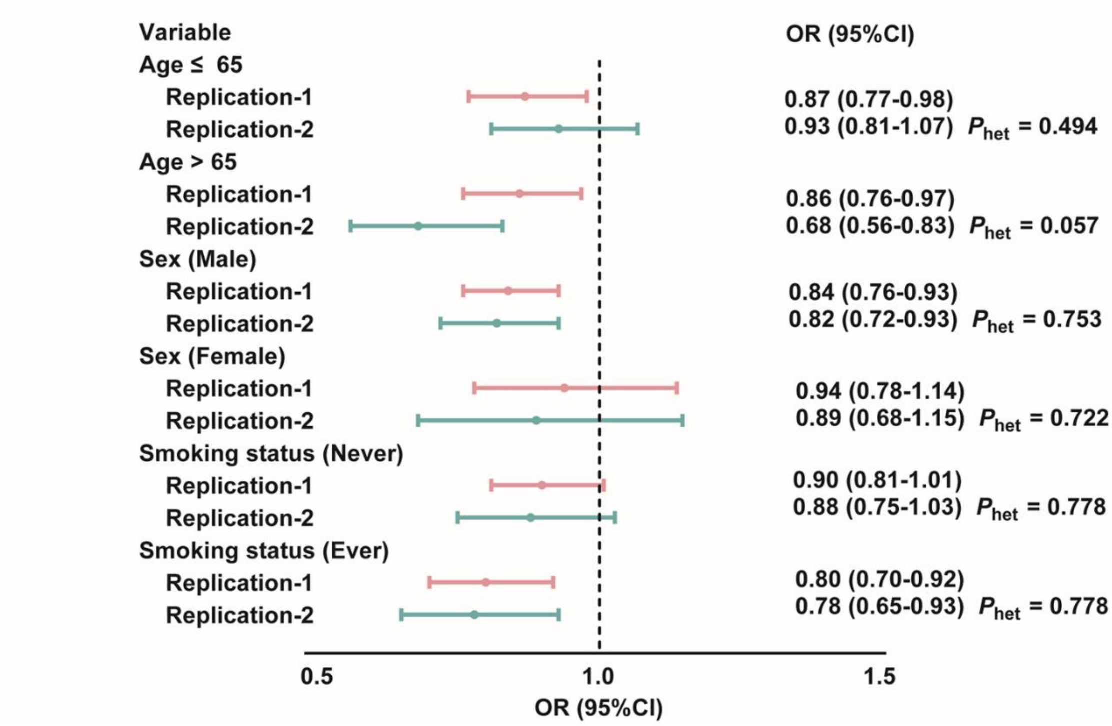

**Figure S5. Power calculations for the sample size.** The red pots show the power of this study.

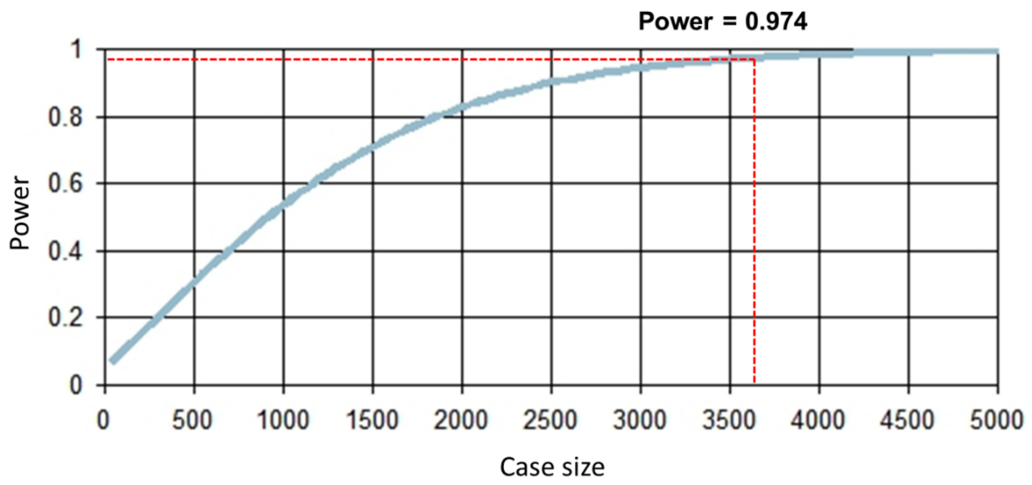

**Figure S6. Characterization of full-length lncRNA *BCCE4* in EJ cell lines by RACE assays.**

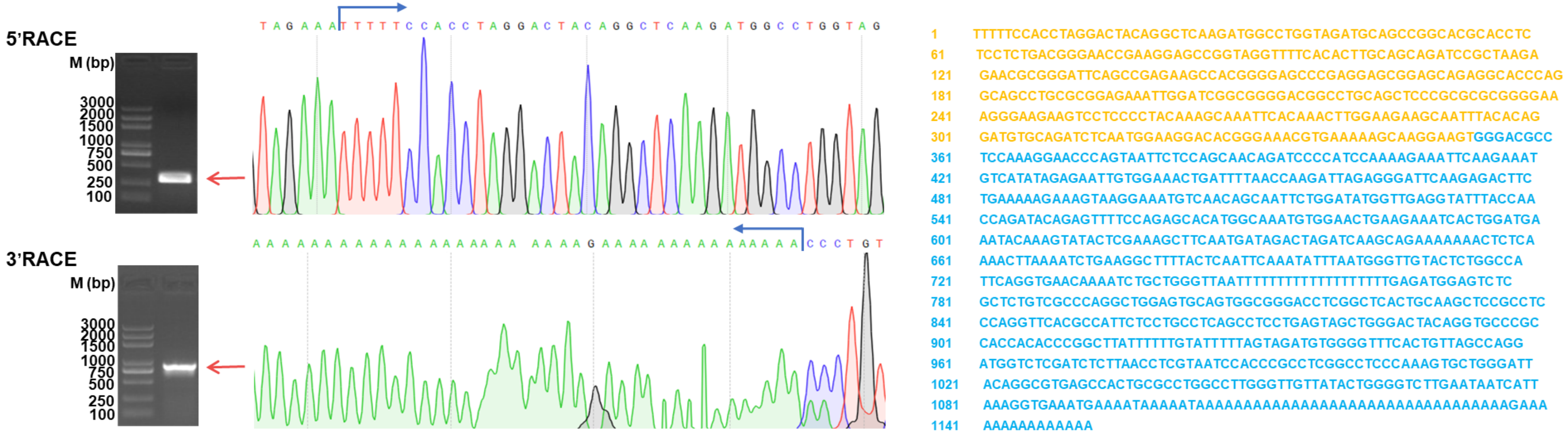

**Figure S7. Characterization of the protein-coding potential for lncRNA *BCCE4*.**

The protein coding potential of lncRNA *BCCE4* as predicted by using ORF finder software.

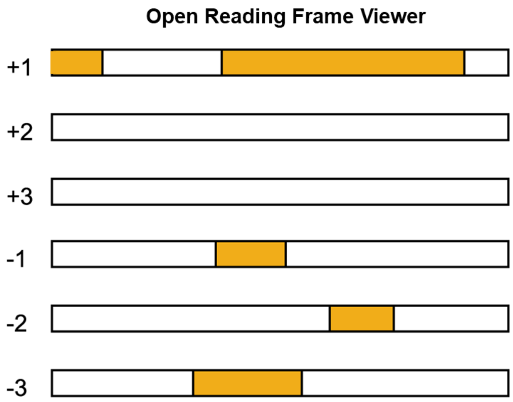


| Frame | Start | Stop | Length（nt/aa） |
|---|---|---|---|
| +1 | <1 | 78 | 78/25 |
| +1 | 262 | 636 | 375/124 |
| -1 | 360 | 253 | 108/35 |
| -2 | 527 | 429 | 99/32 |
| -3 | 385 | 218 | 168/55 |

**Figure S8. Expression of lncRNA *BCCE4* in bladder cancer. (A**) The expression of lncRNA *BCCE4* as detected in patients with bladder cancer either in low or high grade from TCGA database. **(B)** The expression levels of exosomal lncRNA *BCCE4* in plasma from bladder cancer cases (n=22) and cancer-free controls (n=17) as determined by using RT–qPCR. **(C)** Differential expression of lncRNA *BCCE4* was detected in the exosomes purified from SV-HUC-1, EJ and J82 cells. Statistical significance was assessed using two-tailed Student's *t* test. The values represent the mean ± SD. $^{*}P < 0.05$.

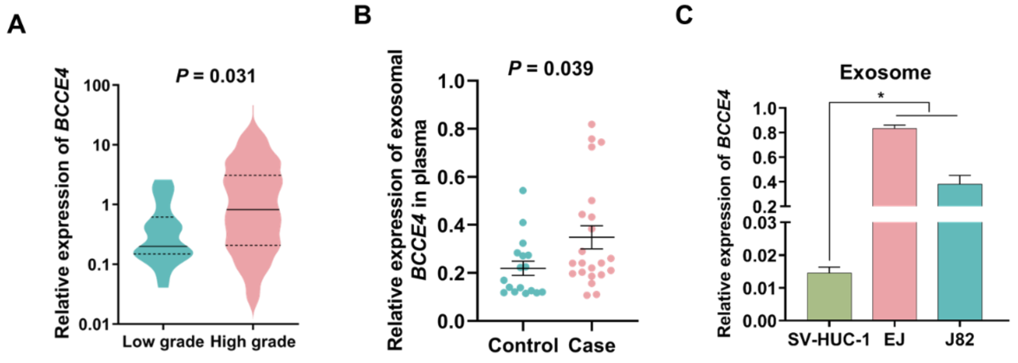

**Figure S9. Expression of lncRNA *BCCE4* in pan-tissues. (A)** The expression of lncRNA *BCCE4* as measured in multiple tumor tissues compared with normal tissues from TCGA database. **(B)** The expression of lncRNA *BCCE4* in pan-tissues obtained from GTEx database.

A

B

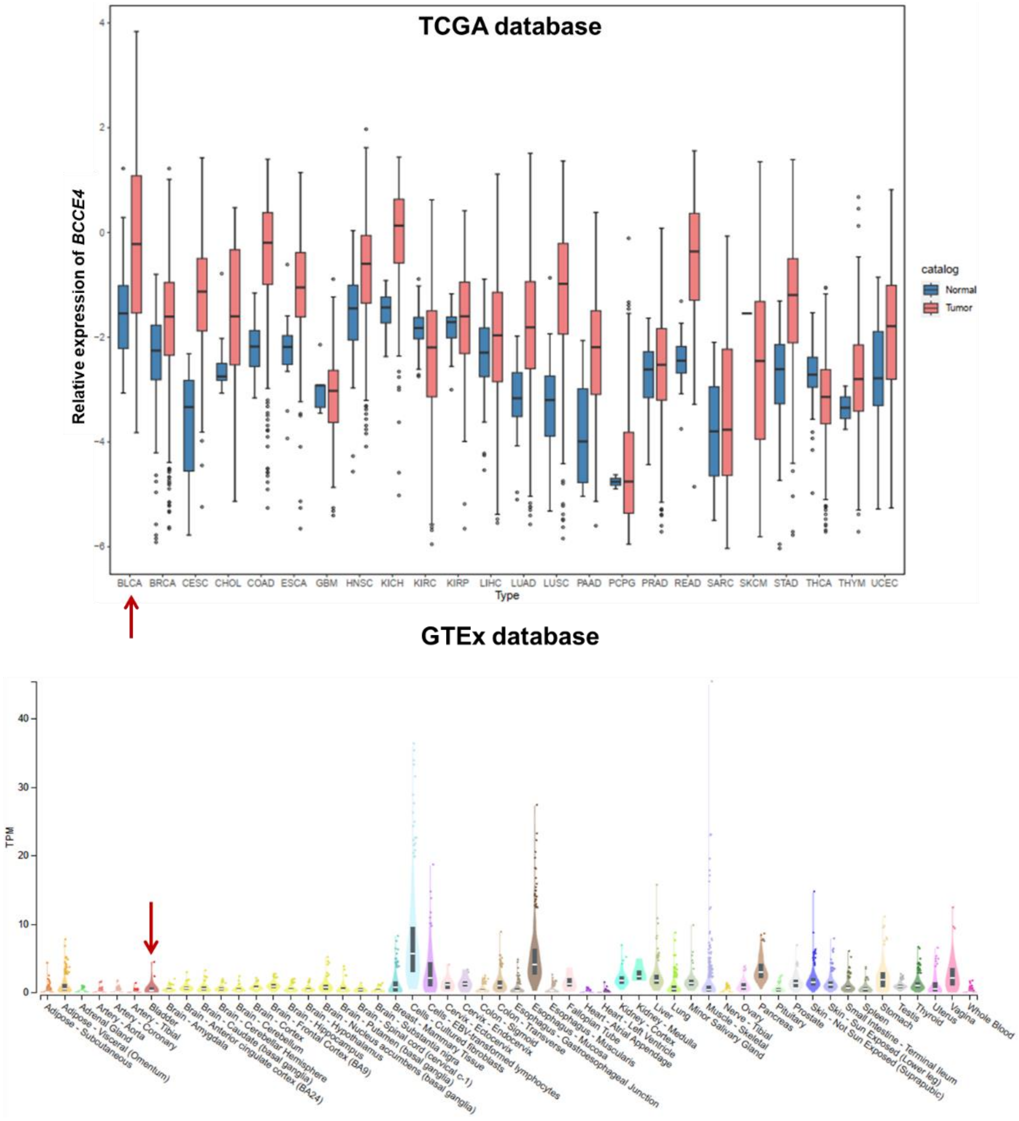

**Figure S10. The effect of lncRNA *BCCE4* knockdown on bladder cancer cellular phenotypes.** The control siRNA or lncRNA *BCCE4* siRNA was transfected into EJ and J82 cells, namely NC and si-*BCCE4*, respectively. **(A)** The expression of lncRNA *BCCE4* in NC or si-*BCCE4* group as measured by RT–qPCR. **(B)** The role of lncRNA *BCCE4* knockdown on the proliferation curves of EJ (left panel) and J82 (right panel) cells as detected by CCK-8 assay. **(C)** The role of lncRNA *BCCE4* knockdown on cell proliferation as measured by using EdU assay. Scale bar, 25 μm. **(D)** The role of lncRNA *BCCE4* knockdown on colony formation ability of EJ (left panel) and J82 (right panel) cells as confirmed by colony formation assay. **(E)** The role of lncRNA *BCCE4* knockdown on the invasion and migration abilities of EJ (upper panel) and J82 (lower panel) cells as determined by a transwell assay. Scale bar, 20 μm. **(F)** The role of lncRNA *BCCE4* knockdown on the cell cycle of EJ (upper panel) and J82 (lower panel) cells as measured by flow cytometry. Statistical significance was assessed using two-tailed Student's *t* test. The values represent the mean ± SD. $^{*}P < 0.05$.

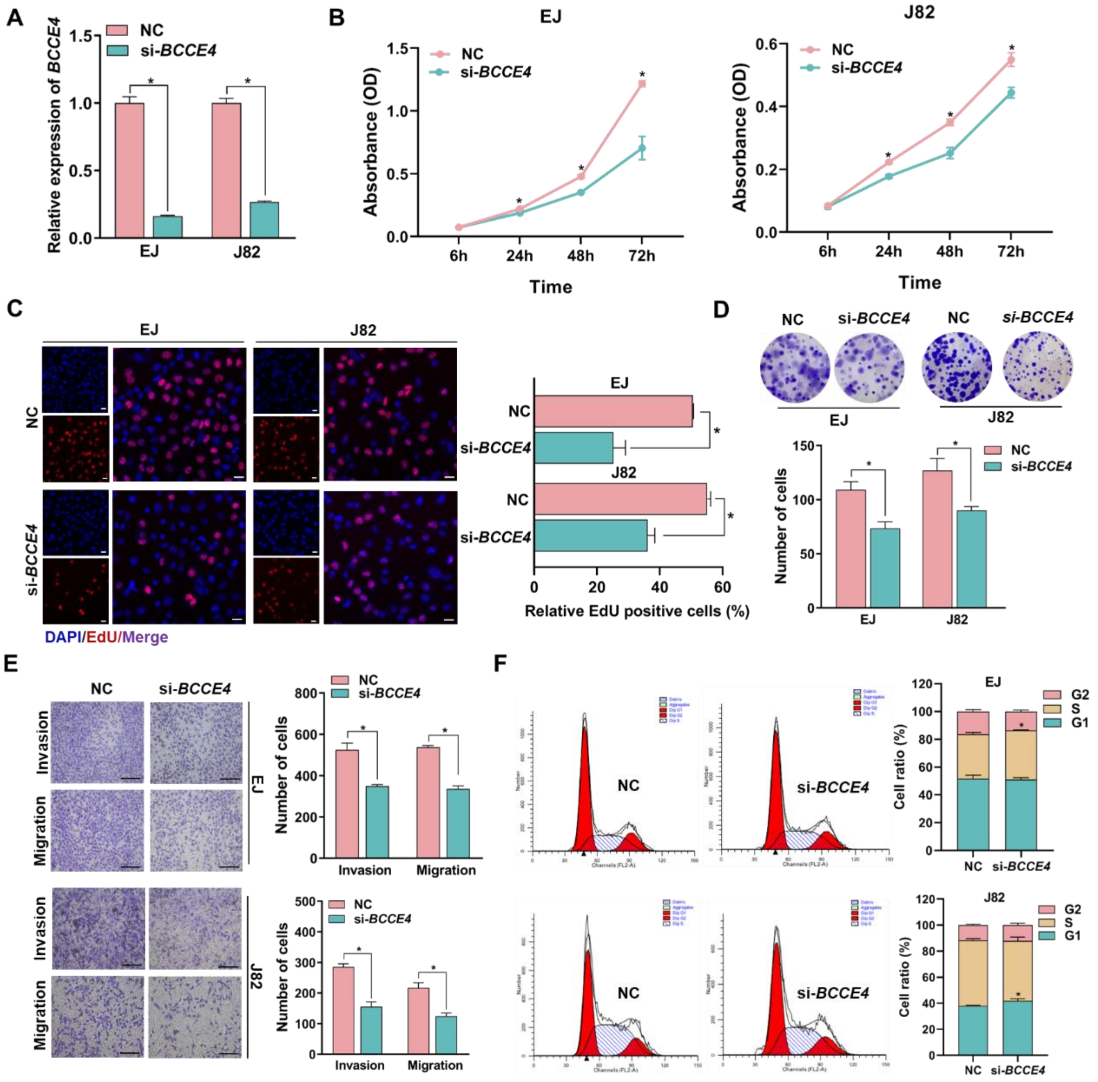
A
NC
si-BCCE4
Relative expression of BCCE4
EJ
J82
B
EJ
J82
Absorbance (OD)
6h
24h
48h
72h
Time
C
NC
si-BCCE4
DAPI/EdU/Merge
Relative EdU positive cells (%)
D
Number of cells
E
Invasion
Migration
Number of cells
F
G2
S
G1
Cell ratio (%)

**Figure S11. Prediction of the effects of rs62483508 G > A change on lncRNA *BCCE4* folding structure.** Predicted folding structures and mountain plots for rs62483508 G **(A)** or rs62483508 A **(B)** in lncRNA *BCCE4* by RNAfold WebServer. Each mountain plot is an x–y graph showing the secondary structures of lncRNA *BCCE4*.

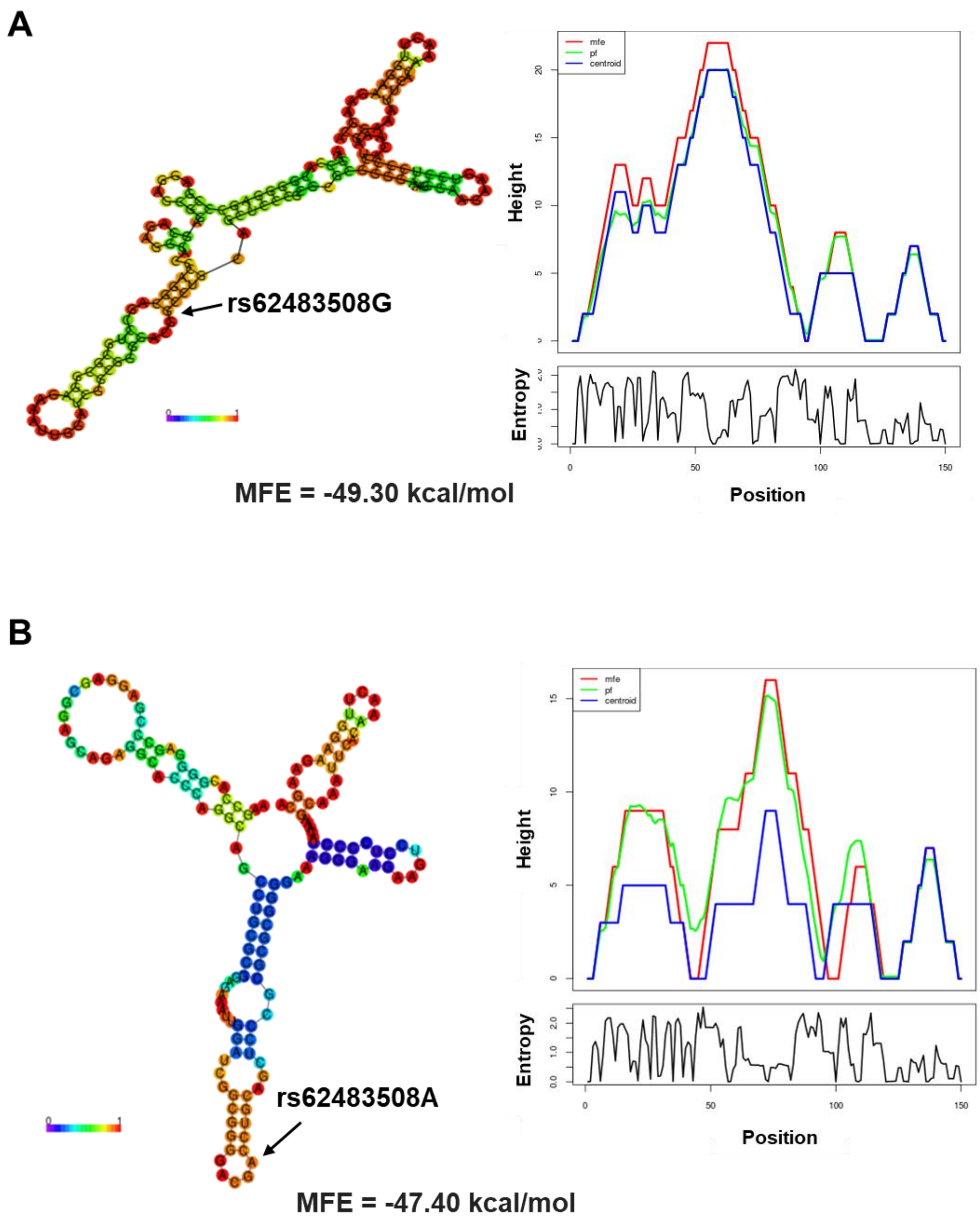

**Figure S12. Prediction of lncRNA *BCCE4* transcription factors.** The predicted preferential binding site of TFAP2A to the lncRNA *BCCE4* rs62483508 by the PROMO **(A)** and JASPAR databases **(B)**. **(C)** The expression of *TFAP2A* in bladder cancer tissues and normal tissues from TCGA database. **(D)** The expression of *TFAP2A* in bladder tumors and normal adjacent tissues from TCGA database. **(E)** Expression correlation analyses for lncRNA *BCCE4* and *TFAP2A* expression levels from TCGA database.

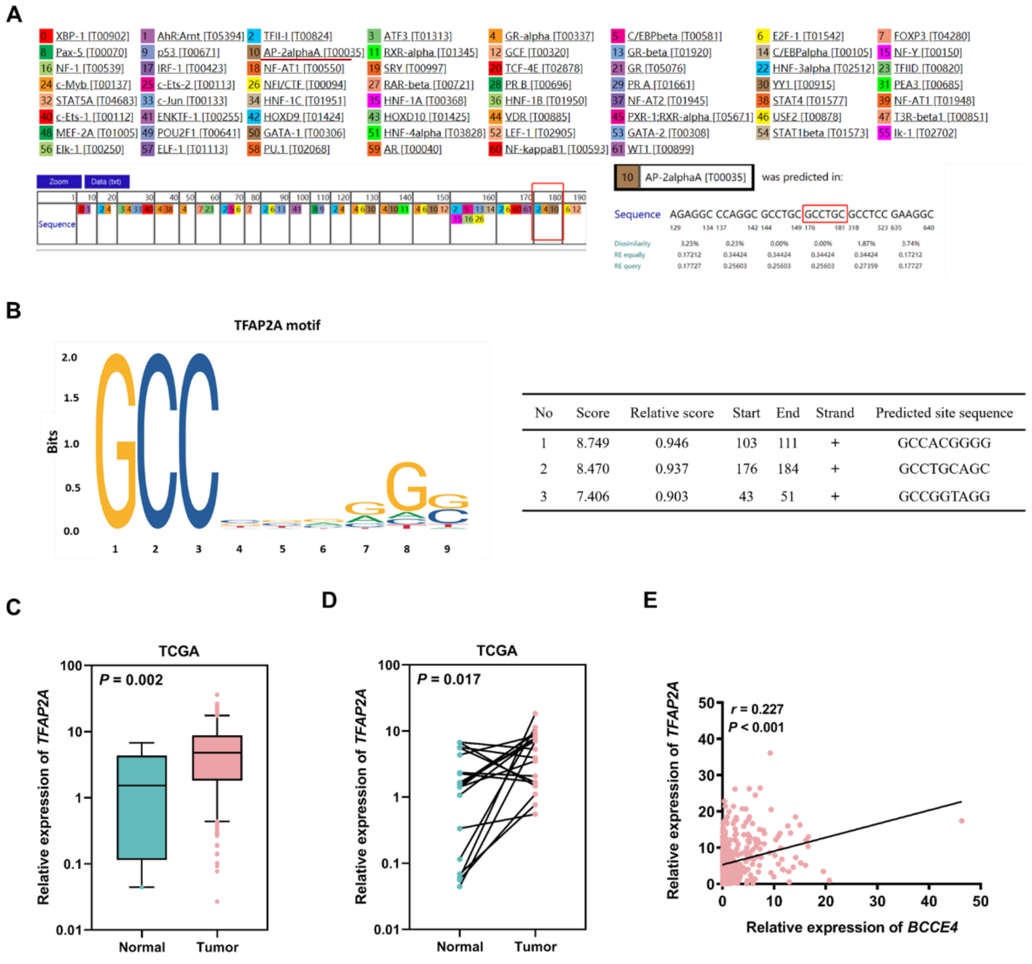

**Figure S13. The genotype of lncRNA *BCCE4* rs62483508 in EJ and J82 cell lines.**

The genotype of rs62483508 as detected in the EJ **(A)** and J82 **(B)** cell lines by using Sanger sequencing. The red arrow represents the genotype of rs62483508 in EJ or J82 cell lines.

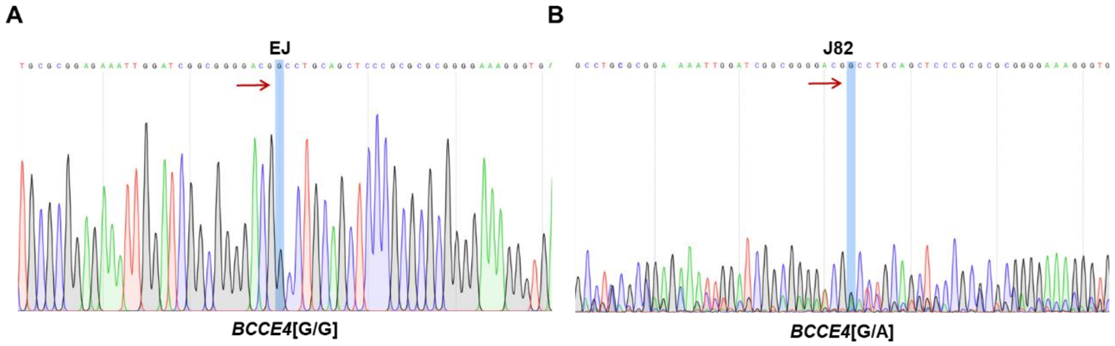

**Figure S14. Determination of the transfection efficiency of *BCCE4*[G] or *BCCE4*[A] stable overexpression.** The rs62483508 G or A lentiviral vector was transfected into EJ and J82 cells, which were designated *BCCE4*[G] and *BCCE4*[A], respectively. Representative fluorescence images of *BCCE4*[G] and *BCCE4*[A] in EJ **(A)** and J82 **(B)** cells. Scale bar, 50 μm. **(C)** The genotype of rs62483508 as detected in EJ cells transfected with *BCCE4*[G] (upper panel) and *BCCE4*[A] (lower panel). **(D)** The genotype of rs62483508 as detected in J82 cells transfected with *BCCE4*[G] (upper panel) and *BCCE4*[A] (lower panel). The red arrow represents the genotype of rs62483508 in EJ and J82 cell lines.

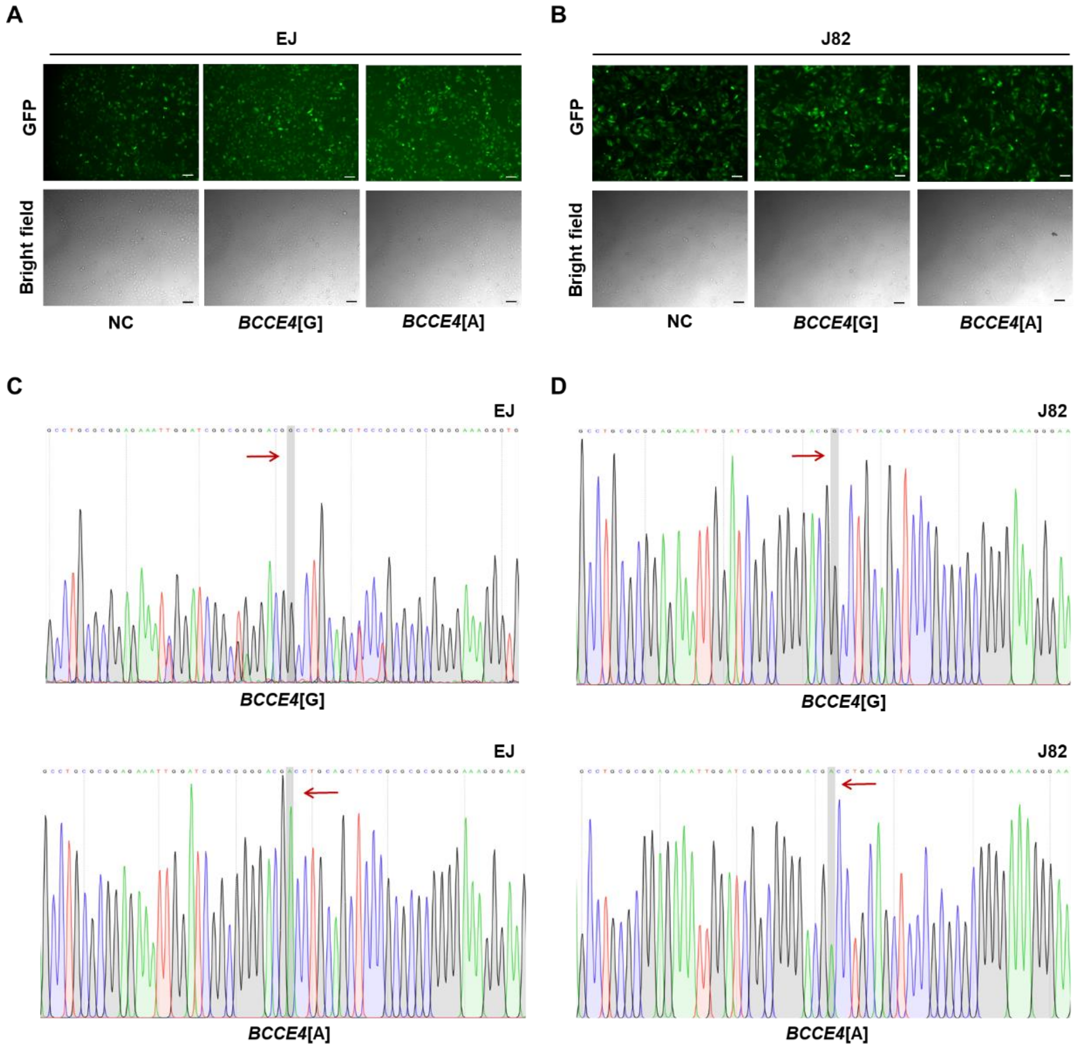

**Figure S15. Biological function of lncRNA *BCCE4* rs62483508 G > A in bladder cancer cellular phenotypes.** The NC, rs62483508 G or A lentiviral vector was transfected into EJ and J82 cells, which were designated NC, *BCCE4*[G] and *BCCE4*[A], respectively. **(A)** The effect of *BCCE4*[G] and *BCCE4*[A] overexpression on cell proliferation as determined by EdU assay. Scale bar, 25 μm. **(B)** The effect of *BCCE4*[G] and *BCCE4*[A] overexpression on the proliferation curves of EJ (upper panel) and J82 (lower panel) cells as determined by CCK-8 assay. **(C)** The effect of *BCCE4*[G] and *BCCE4*[A] overexpression on the invasion and migration abilities in EJ (left panel) and J82 (right panel) cells lines as analyzed by a transwell assay. Statistical significance was assessed using two-tailed Student's *t* test. The values represent the mean ± SD. $^{*}P < 0.05$.

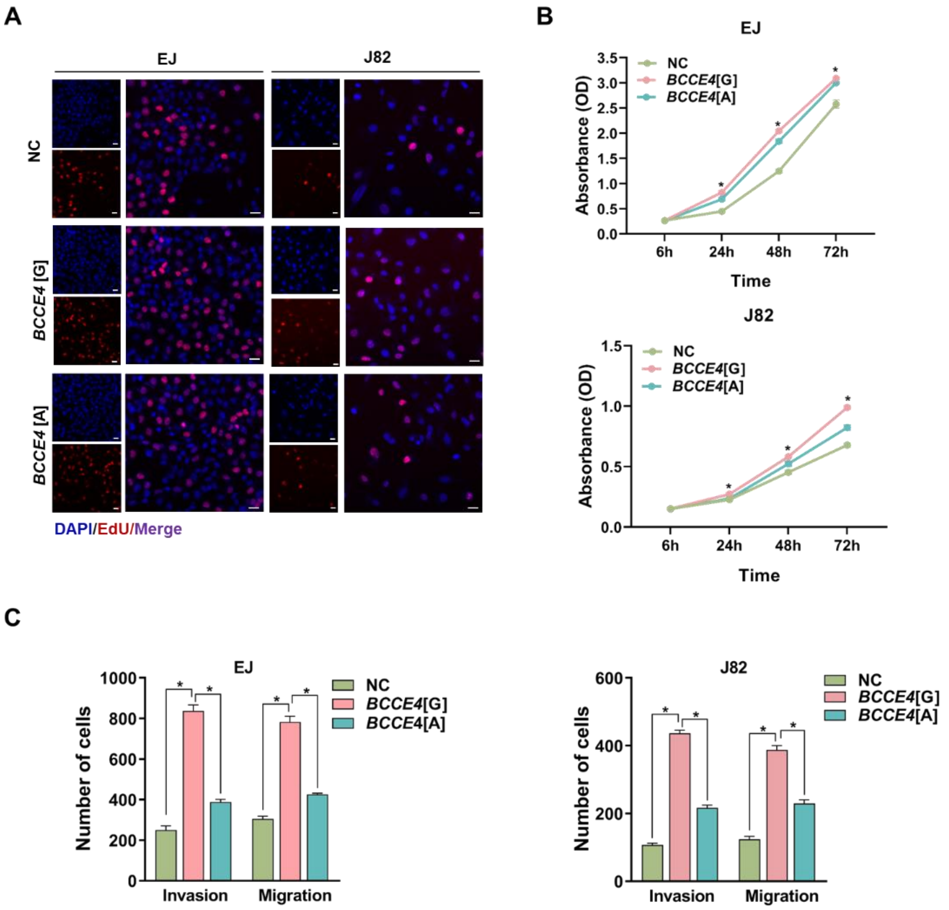

**Figure S16. The cellular localization of lncRNA *BCCE4* in bladder cancer cells.**

FISH analysis representing the cellular location of lncRNA *BCCE4*, 18S and U6 in EJ **(A)** and J82 **(B)** cells. 18S and U6 were used as positive controls for the fractions of cytoplasm and nucleus, respectively. Scale bar, 25μm.

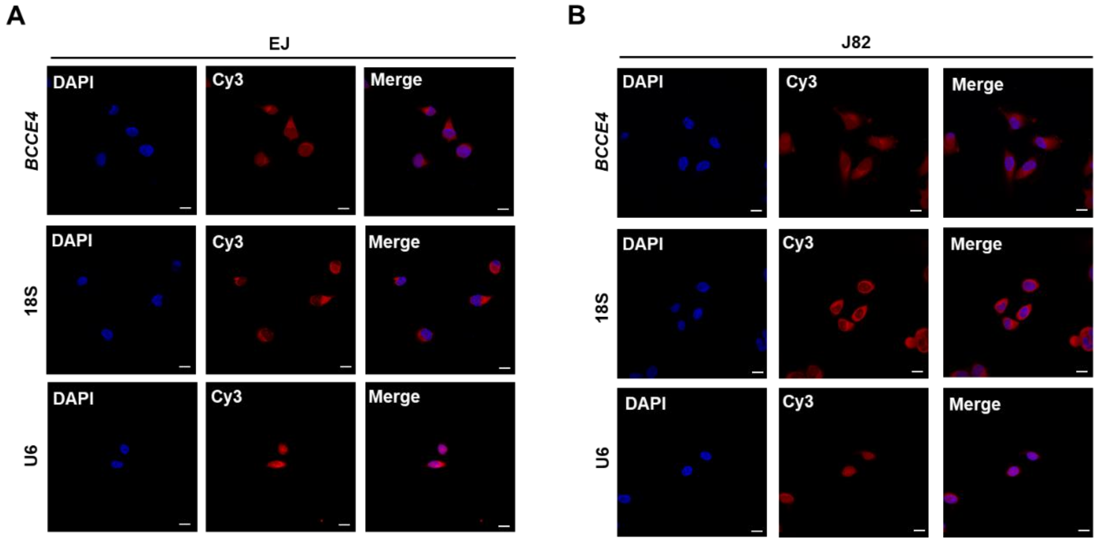

**Figure S17. Expression of AGO2 in bladder cancer cells transfected with si-*BCCE4*.** The control siRNA or lncRNA *BCCE4* siRNA was transfected into EJ and J82 cells, namely, NC and si-*BCCE4*, respectively. The protein levels of AGO2 in NC and si-*BCCE4* groups in EJ **(A)** and J82 **(B)** cell lines as detected by Western blotting. Statistical significance was assessed using two-tailed Student's *t* test. The values represent the mean ± SD.

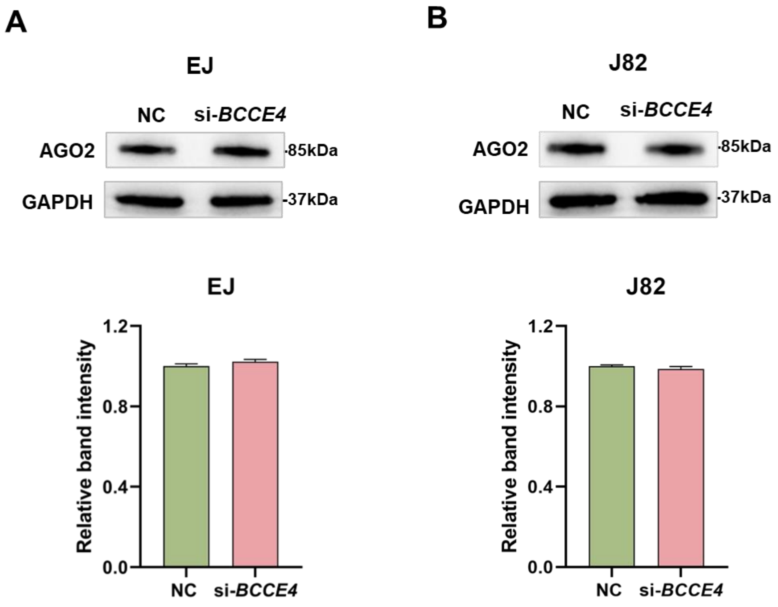

**Figure S18. Function relevance of the lncRNA *BCCE4* variant.** The line chart compares the slope difference of the modification for relatively luciferase activity between the constructs containing *BCCE4*[G] and *BCCE4*[A] in EJ **(A)** and J82 **(B)** cells transfected with different concentrations (20, 40 and 80 pmol) of miR-328-3p mimic. Statistical significance was assessed using two-tailed Student's *t* test. The values represent the mean ± SD. $^{*}P < 0.05$.

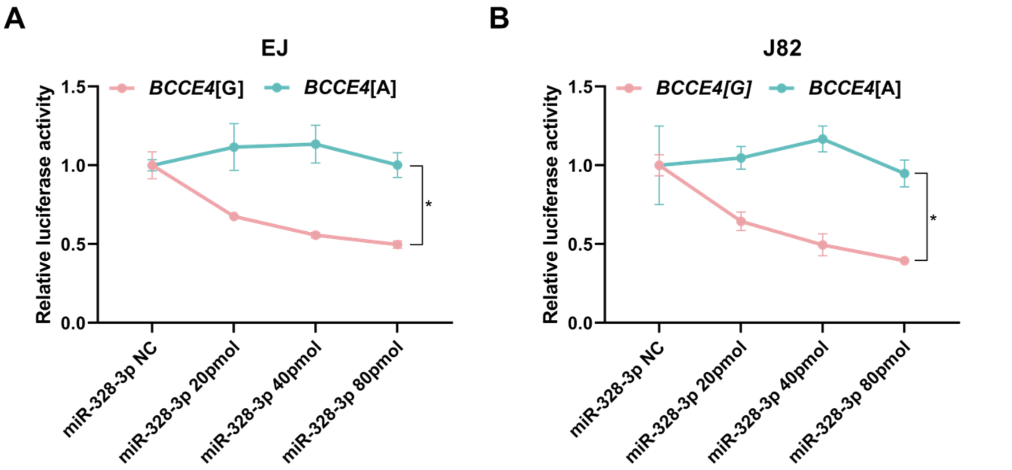

**Figure S19. The effect of miR-328-3p on lncRNA *BCCE4* expression levels in an allele-specific manner.** Relative expression levels of miR-328-3p in EJ **(A)** and J82 **(B)** cells transfected with miR-328-3p mimic (80 pmol) or inhibitor. **(C-D)** The effect of different doses (20, 40 and 80 pmol) of miR-328-3p mimic on lncRNA *BCCE4* expression levels in bladder cancer cell lines with different lncRNA *BCCE4* genotype. **(E-F)** The effect of 80 pmol of miR-328-3p mimic or inhibitor on lncRNA *BCCE4* expression levels in bladder cancer cell lines with different lncRNA *BCCE4* genotype. Statistical significance was assessed using two-tailed Student's *t* test. The values represent the mean ± SD. $^{*}P < 0.05$.

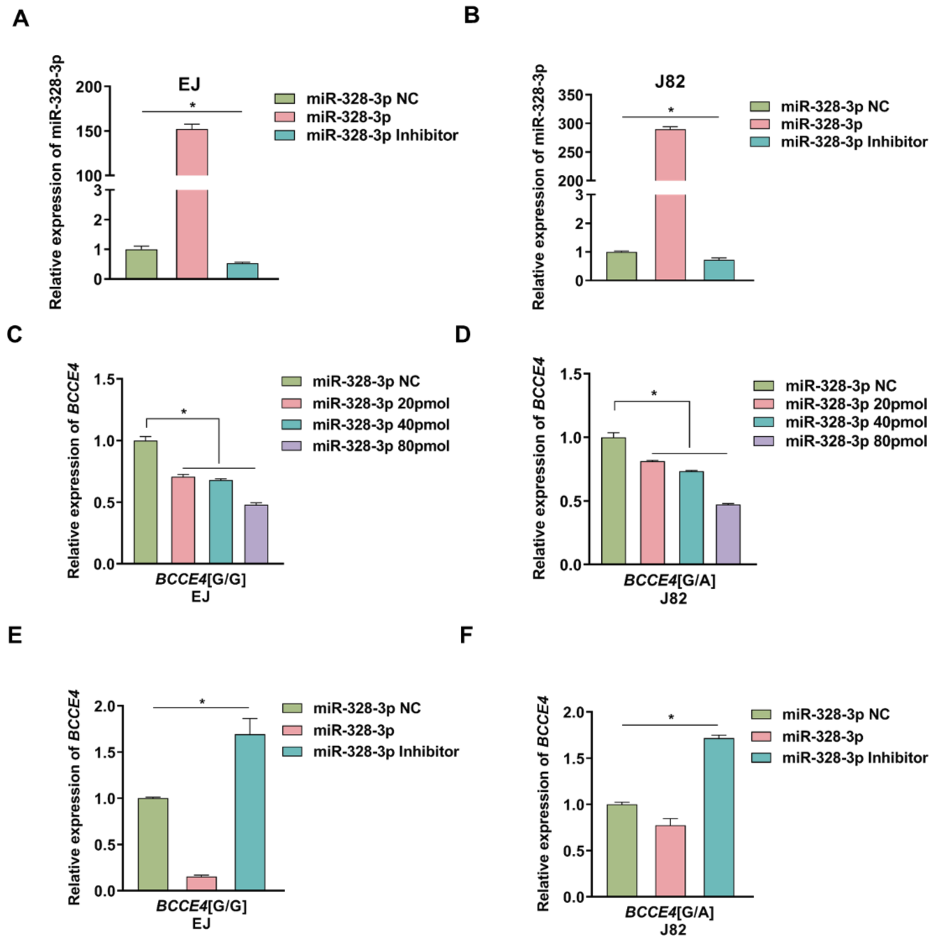

**Figure S20. The interaction of between lncRNA *BCCE4* and miR-328-3p. (A)** The colocalization assessment of lncRNA *BCCE4* (red) and miR-328-3p (green) colocalization in EJ and J82 cells. Scale bar, 20 μm. **(B)** MS2-RIP showing the interaction of between MS2-lncRNA *BCCE4* and the endogenous miR-328-3p. Statistical significance was assessed using two-tailed Student's *t* test. The values represent the mean ± SD. $^{*}P < 0.05$.

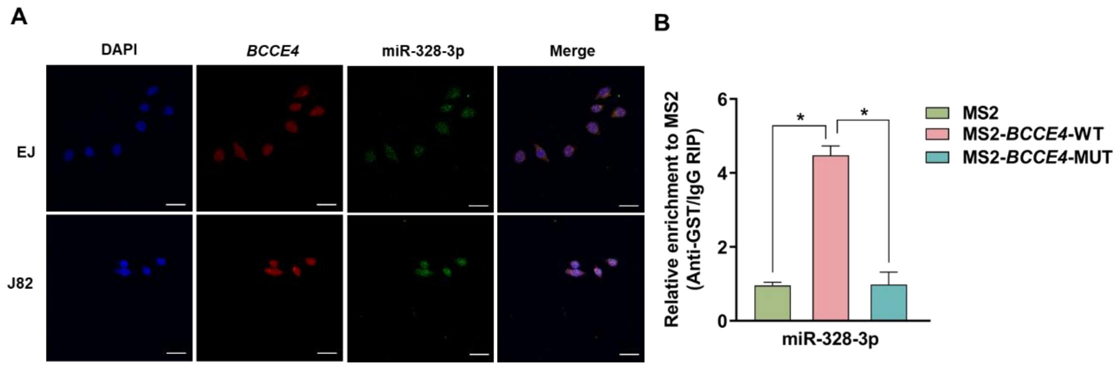

**Figure S21. Genome-wide gene expression variation after miR-328-3p modulation.** EJ cells were transfected with miR-328-3p mimic or NC, namely miR-328-3p and miR-328-3p NC, respectively. **(A)** Heatmap of significantly different gene mRNA levels (|fold change| > 1.8, $P < 0.05$) as detected by RNA sequencing in miR-328-3p and miR-328-3p NC group. **(B)** Heatmap of significantly different protein levels (|fold change| > 1.2, $P < 0.05$) as detected by tandem mass tag (TMT) in miR-328-3p and miR-328-3p NC group. **(C)** Venn diagram showing the overlapping genes/proteins between transcriptomics and proteomics.

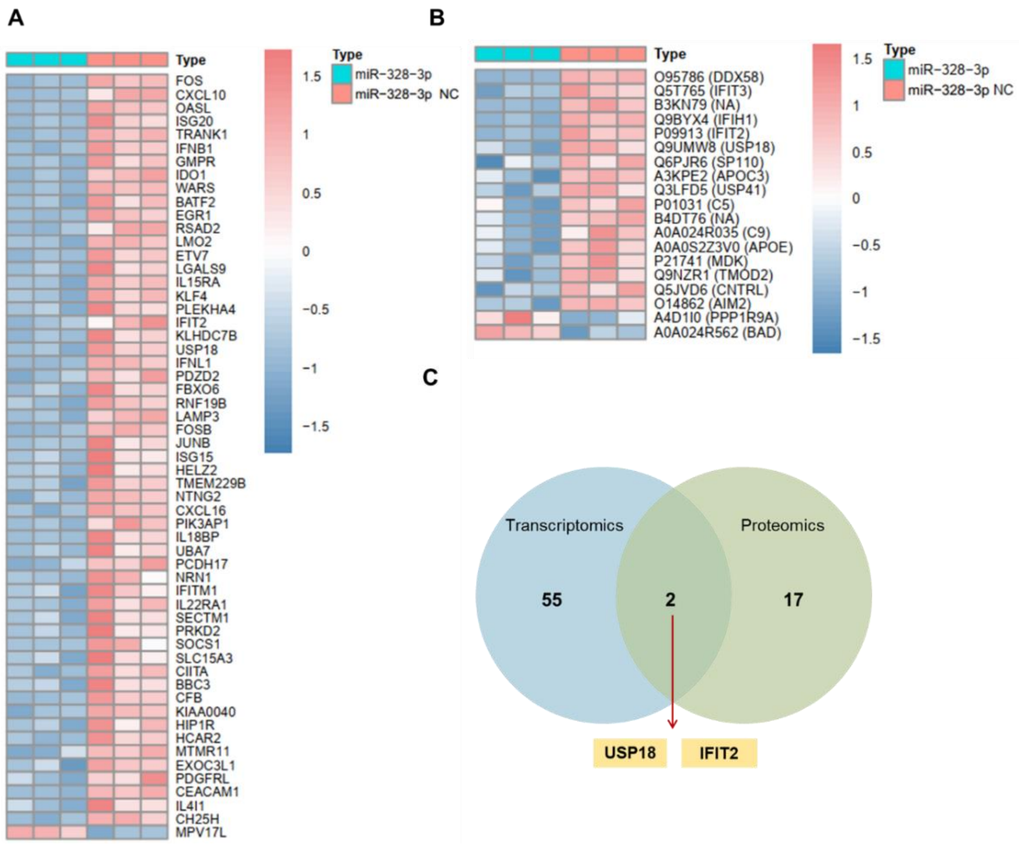

**Figure S22.** ***USP18*** **and** ***IFIT2*** **expression patterns in bladder cancer tissues. (A)** The expression of *USP18* in 29 pairs of bladder tumors and normal adjacent tissues from NJBC dataset. **(B)** The expression of *USP18* in 19 pairs of bladder tumors and normal adjacent tissues from TCGA database. **(C)** The expression of *USP18* in bladder cancer tissues and normal tissues from TCGA database. **(D)** The expression of *IFIT2* in 29 pairs of bladder tumors and normal adjacent tissues from NJBC dataset. **(E)** The expression of *IFIT2* in 19 pairs of bladder tumors and normal adjacent tissues from TCGA database. **(F)** The expression of *IFIT2* in bladder cancer tissues and normal tissues from TCGA database. Statistical significance was assessed using two-tailed or paired Student's *t* test.

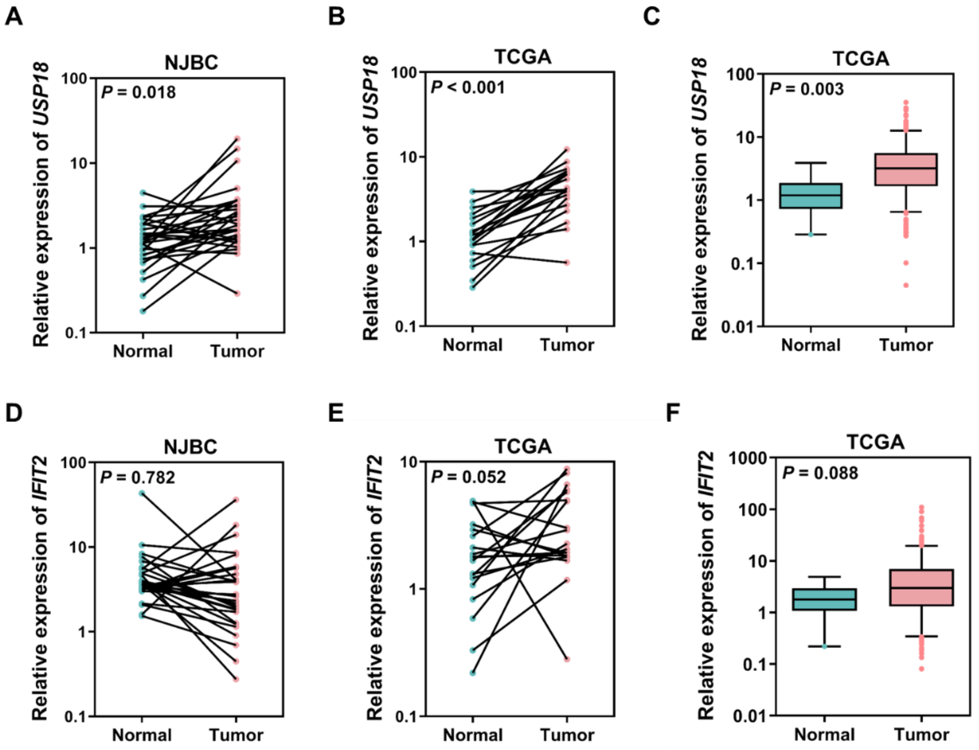

**Figure S23. The expression of USP18 in pan-tissues and bladder cancer tissues. (A-C)** The expression levels of *USP18* in bladder cancer tissues and normal tissues from GSE13507, GSE65635 and GSE7476. **(D-E)** The levels of USP18 in pan-tissues from HPA and FANTOM5 datasets.

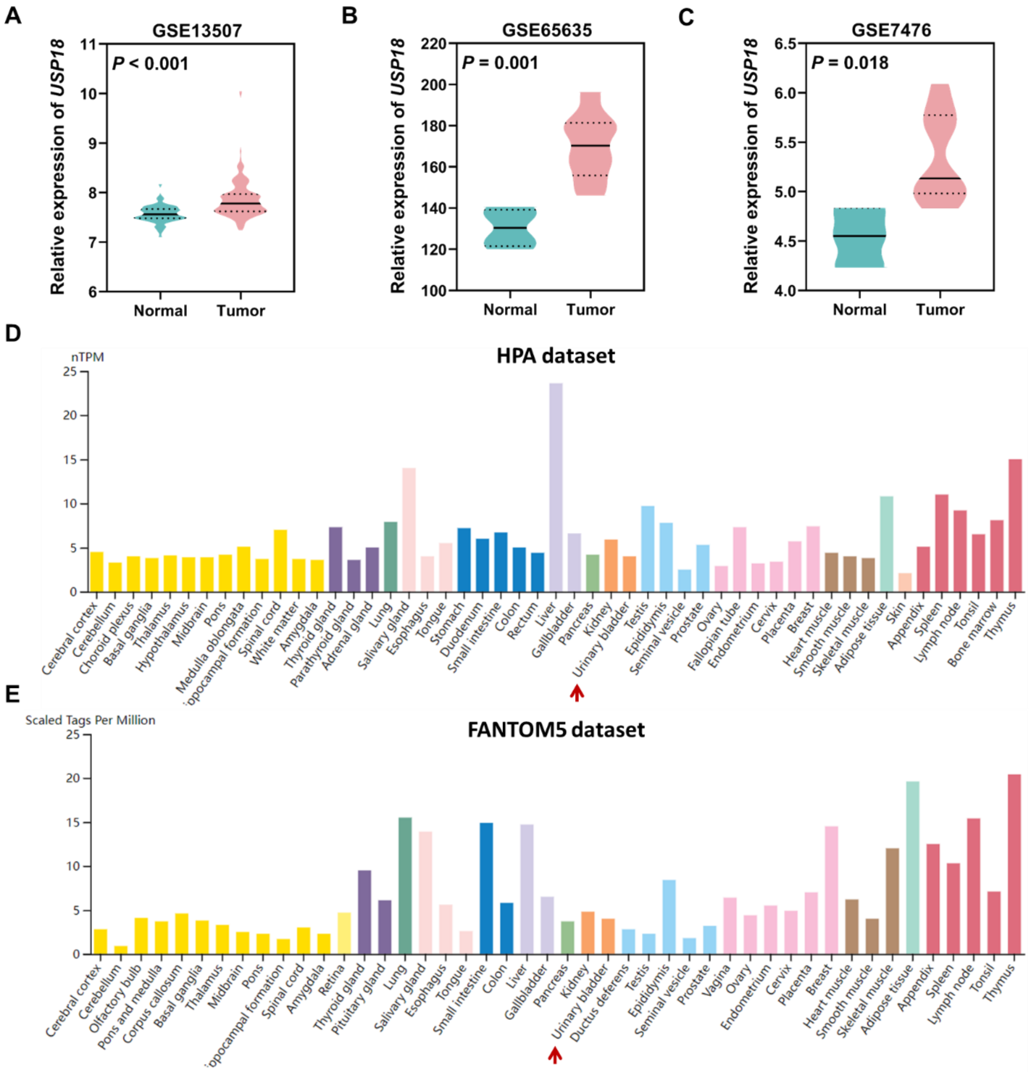

**Figure S24. Identification of the target gene of miR-328-3p. (A)** The predicted wild-type (WT) seed sequence of USP18 complementary to miR-328-3p and investigator-designed mutated (MUT) seed sequences of USP18. **(B)** The expression of *USP18* in EJ cells transfected with 80 pmol of miR-328-3p mimic or inhibitor. Statistical significance was assessed using two-tailed Student's *t* test. The values represent the mean ± SD. $^{*}P < 0.05$. **(C)** The correction between lncRNA *BCCE4* and *USP18* levels in bladder cancer tissues from TCGA database. Statistical significance was assessed using Spearman's correlation analysis. **(D)** The relative enrichment of USP18 in EJ cells transfected with lncRNA *BCCE4* overexpression vectors as measured by using RNA pull-down assays and Western blotting.

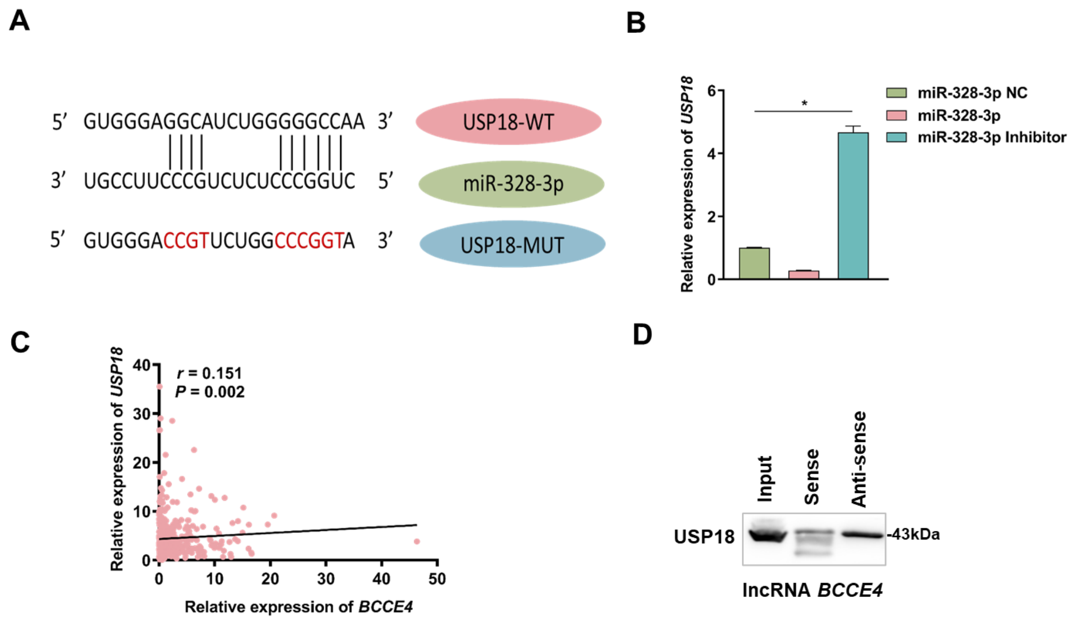

**Figure S25. The effect of miR-328-3p regulation by lncRNA *BCCE4* rs62483508 G > A on bladder cancer cellular phenotype.** The rs62483508 G or A lentiviral vector was transfected into EJ cells, namely *BCCE4*[G] and *BCCE4*[A]**,** respectively. The miR-328-3p mimic (80 pmol) were transfected into *BCCE4*[G] cells, which were designated *BCCE4*[G]+miR-328-3p. **(A)** The cell proliferation of the indicated cells as determined by EdU assay. Scale bar, 25 μm. **(B)** The invasion and migration abilities of the indicated cells as measured by using a transwell assay. Scale bar, 20 μm. Statistical significance was assessed using two-tailed Student's *t* test. The values represent the mean ± SD. $^{*}P < 0.05$.

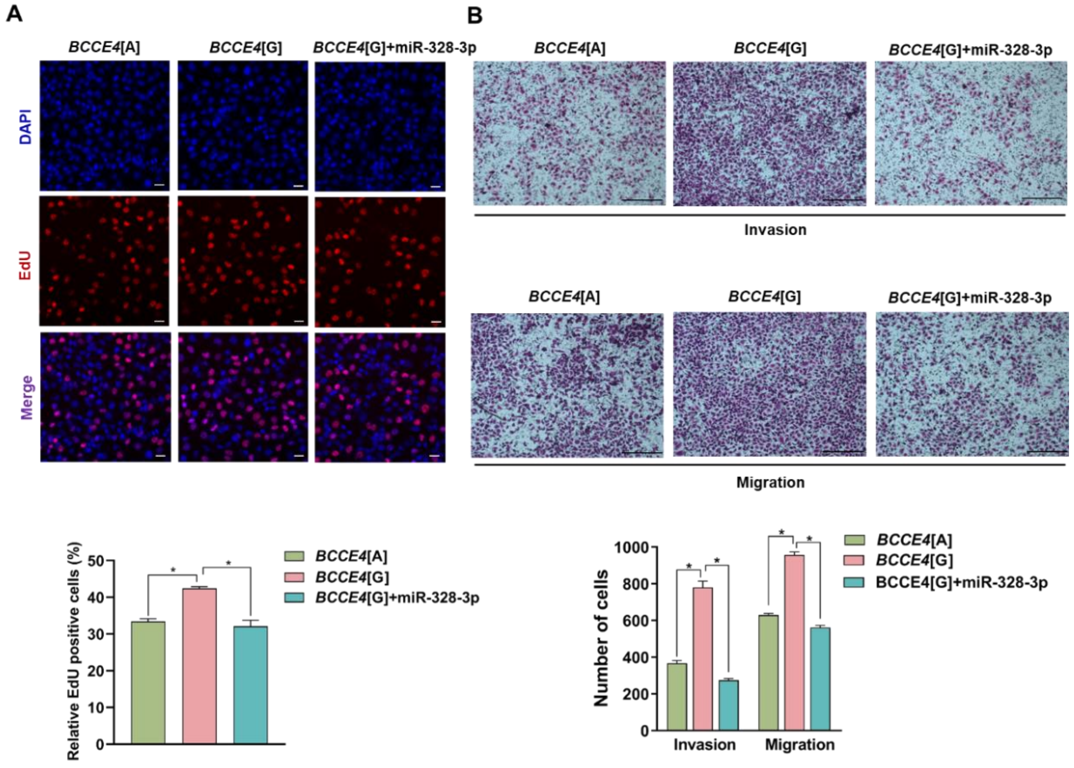

**Figure S26. The effect of USP18 regulation by lncRNA *BCCE4* rs62483508 G > A on bladder cancer cellular phenotype.** The rs62483508 G or A lentiviral vector was transfected into EJ cells, namely, *BCCE4*[G] and *BCCE4*[A], respectively. The USP18 overexpression plasmids were transfected into *BCCE4*[A] cells, which were designated *BCCE4*[A]+USP18. **(A)** The cell proliferation of the indicated cells as determined by EdU assay. Scale bar, 25 μm. **(B)** The invasion and migration abilities of the indicated cells as measured by using a transwell assay. Scale bar, 20 μm. Statistical significance was assessed using two-tailed Student's *t* test. The values represent the mean ± SD. $^{*}P < 0.05$.

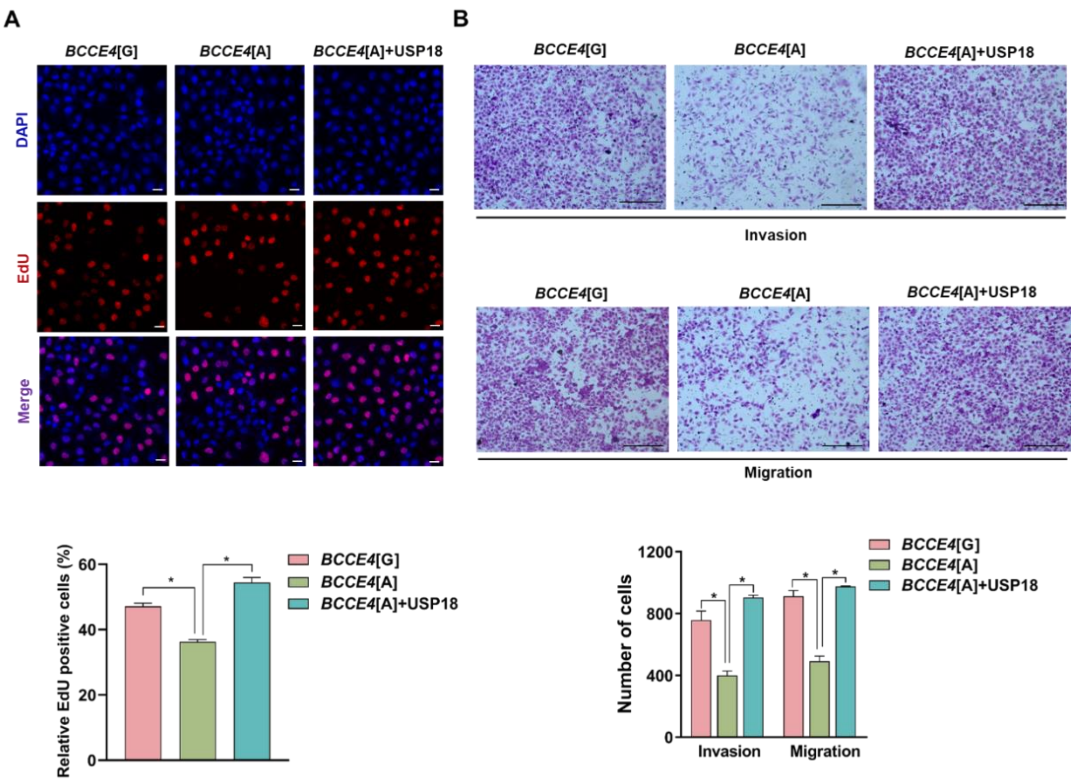

**Figure S27. Biological function of lncRNA *BCCE4* rs62483508 G > A in lncRNA *BCCE4* knockout bladder cancer cell lines.** Negative control, *BCCE4*[G] or *BCCE4*[A] vectors were transfected into the lncRNA *BCCE4* knockout (*BCCE4*-KO) bladder cancer cell lines. **(A)** The expression of lncRNA *BCCE4* in EJ cells before (NC) or after CRISPR/Cas9-mediated knockout of lncRNA *BCCE4* (*BCCE4*-KO). **(B-C)** The levels of USP18 and PD-L1 in the *BCCE4*-KO, *BCCE4*[G] or *BCCE4*[A] groups as measured by Western blotting. **(D-E)** Cell proliferation as measured by using the EdU assay. Scale bar, 25 μm. The invasion **(F)** and migration **(G)** abilities of the indicated cells as measured by using a transwell assay. Scale bar, 20 μm. Statistical significance was assessed using two-tailed Student's *t* test. The values represent the mean ± SD. $^{*}P < 0.05$.

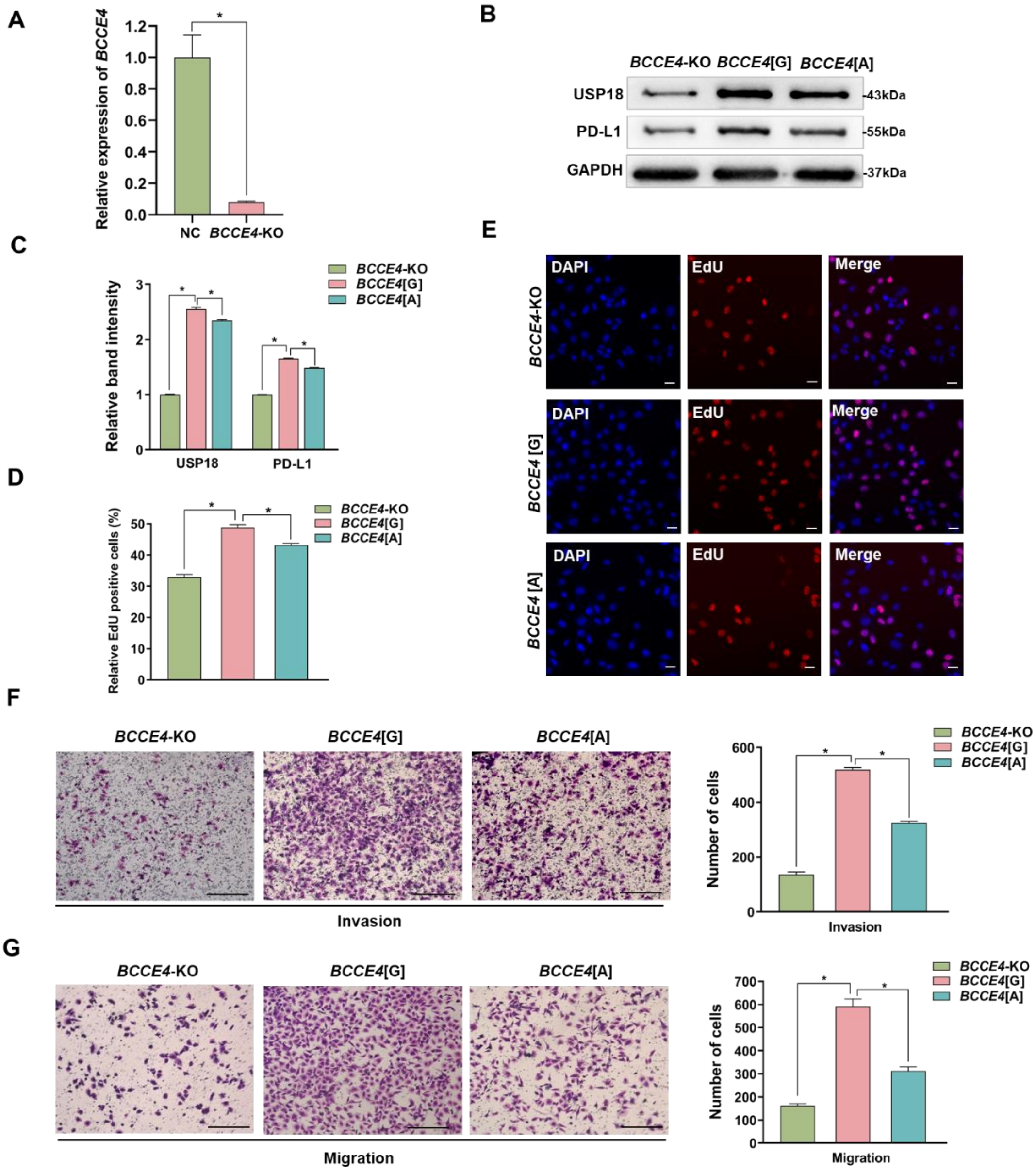
A
Relative expression of BCCE4
1.2
1.0
0.8
0.6
0.4
0.2
0.0
NC
BCCE4-KO
B
BCCE4-KO
BCCE4[G]
BCCE4[A]
USP18
PD-L1
GAPDH
-43kDa
-55kDa
-37kDa
C
Relative band intensity
USP18
PD-L1
D
Relative EdU positive cells (%)
E
DAPI
EdU
Merge
BCCE4-KO
BCCE4 [G]
BCCE4 [A]
F
Invasion
Number of cells
G
Migration

**Figure S28. The correlation between lncRNA *BCCE4*, USP18 and PD-L1 in bladder cancer. (A)** Correlation analysis was performed between *USP18* and *PD-L1* expression levels in bladder cancer tissues from TCGA database. **(B)** Correlation analysis was performed between lncRNA *BCCE4* and *PD-L1* expression levels in bladder cancer tissues from TCGA database. **(C)** The protein levels of USP18 in J82 cells transfected with lncRNA *BCCE4* overexpression plasmids (lncRNA *BCCE4*) or miR-328-3p mimic (miR-328-3p) as detected by Western blotting. Statistical significance was assessed using two-tailed Student's *t* test. The values represent the mean ± SD. $^{*}P < 0.05$. **(D)** Representative fluorescence images of lncRNA *BCCE4*, USP18 and PD-L1 in bladder cancer tissues (n = 16), as obtained by FISH. Scale bars, 100 μm (top) and 20 μm (bottom). Correlation analysis was performed to determine the relationship between the intensity of lncRNA *BCCE4* and USP18 **(E)**, the intensity of lncRNA *BCCE4* and PD-L1 **(F)**, or the intensity of USP18 and PD-L1 (**G**). Statistical significance was assessed using Spearman's correlation analysis.

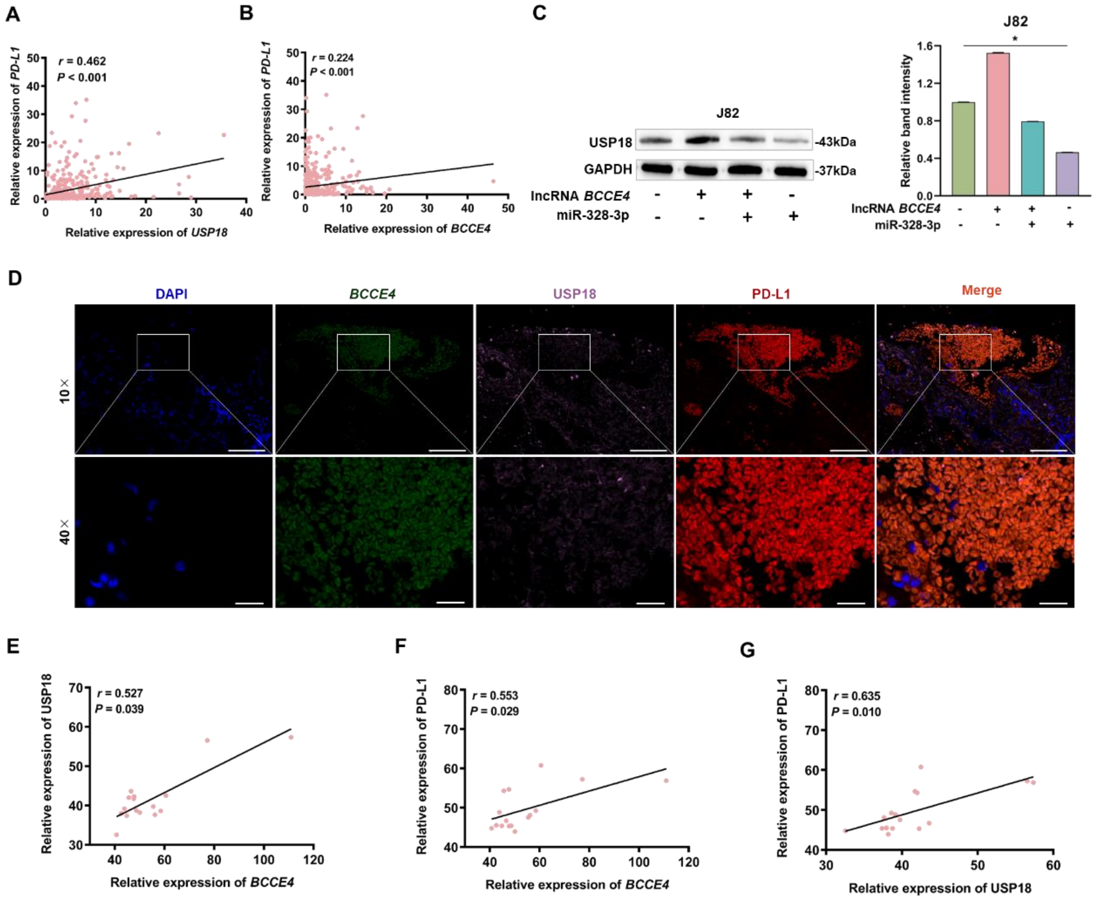
A
r = 0.462
P < 0.001
Relative expression of PD-L1
Relative expression of USP18
B
r = 0.224
P < 0.001
Relative expression of PD-L1
Relative expression of BCCE4
C
J82
USP18
GAPDH
-43kDa
-37kDa
lncRNA BCCE4
miR-328-3p
Relative band intensity
D
DAPI
BCCE4
USP18
PD-L1
Merge
10×
40×
E
r = 0.527
P = 0.039
Relative expression of USP18
Relative expression of BCCE4
F
r = 0.553
P = 0.029
Relative expression of PD-L1
Relative expression of BCCE4
G
r = 0.635
P = 0.010
Relative expression of PD-L1
Relative expression of USP18

**Figure S29. Effect of USP18 on PD-L1 stability. (A)** The stability of PD-L1 in EJ cells when lncRNA *BCCE4* was knocked down as determined by treatment with 20 μmol/L CHX for different times as indicated. **(B)** The protein levels of PD-L1 in EJ cells transfected with UBE1L overexpression plasmids (UBE1L over) and control plasmids (NC) as detected by Western blotting. Statistical significance was assessed using two-tailed Student's *t* test. The values represent the mean ± SD. $^{*}P < 0.05$. Immunoprecipitation (IP) with an anti-V5-USP18 or control lgG followed by immunoblotting (IB) with a second anti-V5-USP18 antibody **(C)** or an anti-HA-ISG15 antibody **(D)**. Immunoprecipitation (IP) with an anti-HA-ISG15 or control lgG followed by immunoblotting (IB) with a second anti-HA-ISG15 antibody **(E)** or an anti-Flag-PD-L1 antibody **(F)**. USP18 could remove ISG15 from the PD-L1 protein following anti-Flag immunoprecipitation of Flag-tagged PD-L1 protein followed by anti-Flag **(G)** or anti-ISG15 IB antibody **(H)** to detect PD-L1.

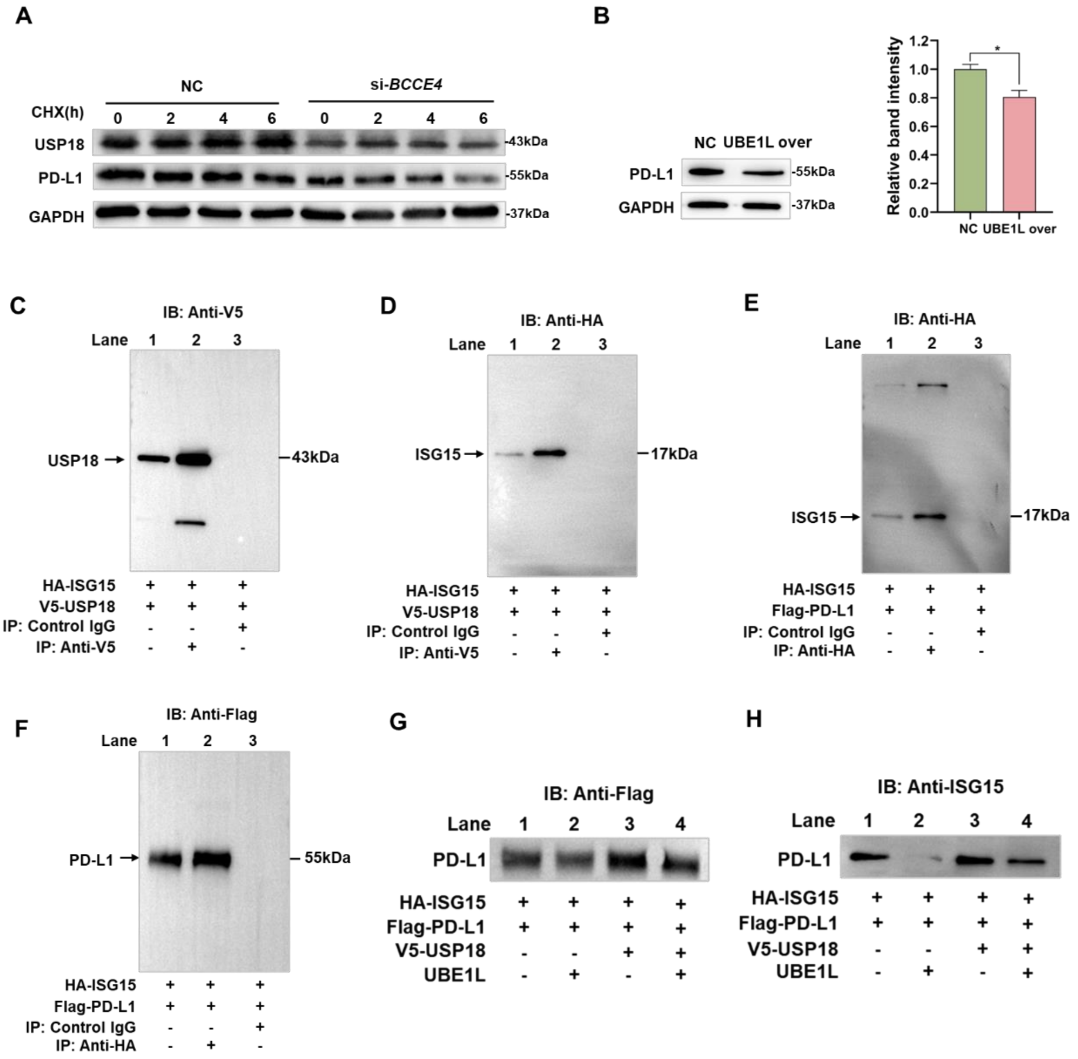
A
NC
si-BCCE4
CHX(h)
0
2
4
6
USP18
PD-L1
GAPDH
-43kDa
-55kDa
-37kDa
B
NC UBE1L over
PD-L1
GAPDH
-55kDa
-37kDa
Relative band intensity
1.2
1.0
0.8
0.6
0.4
0.2
0.0
*
NC UBE1L over
C
IB: Anti-V5
Lane 1 2 3
USP18
43kDa
HA-ISG15 + + +
V5-USP18 + + +
IP: Control IgG - - +
IP: Anti-V5 - + -
D
IB: Anti-HA
Lane 1 2 3
ISG15
17kDa
HA-ISG15 + + +
V5-USP18 + + +
IP: Control IgG - - +
IP: Anti-V5 - + -
E
IB: Anti-HA
Lane 1 2 3
ISG15
17kDa
HA-ISG15 + + +
Flag-PD-L1 + + +
IP: Control IgG - - +
IP: Anti-HA - + -
F
IB: Anti-Flag
Lane 1 2 3
PD-L1
55kDa
HA-ISG15 + + +
Flag-PD-L1 + + +
IP: Control IgG - - +
IP: Anti-HA - + -
G
IB: Anti-Flag
Lane 1 2 3 4
PD-L1
HA-ISG15 + + + +
Flag-PD-L1 + + + +
V5-USP18 - - + +
UBE1L - + - +
H
IB: Anti-ISG15
Lane 1 2 3 4
PD-L1
HA-ISG15 + + + +
Flag-PD-L1 + + + +
V5-USP18 - - + +
UBE1L - + - +

**Figure S30. Effect of lncRNA *BCCE4* on T-cell cytotoxicity via the PD-L1/PD-1 interaction.** The lncRNA *BCCE4* overexpression plasmids or si-USP18 were transfected into EJ cells, namely, *BCCE4* over or si-USP18, respectively. **(A)** Binding of PD-1 with cell surface PD-L1 of EJ cells when lncRNA *BCCE4* was overexpressed or USP18 was knocked down. Scale bar, 25 μm. **(B)** *BCCE4* over or si-USP18 cells were cocultured with activated Jurkat T cells at the indicated E/T ratios for 24 h. Scale bar, 20 μm. **(C)** *BCCE4* over or si-USP18 cells were cocultured with activated PBMCs at the indicated E/T ratios for 24 h, and the relative cytotoxicity was measured by lactate dehydrogenase (LDH) assays. **(D)** The rs62483508 G lentiviral vector was transfected into EJ cells, which were designated *BCCE4*[G]. *BCCE4*[G] cells incubated with anti-PD-L1 antibody were then cocultured with activated Jurkat T cells at the indicated E/T ratios for 24 h. Scale bar, 20 μm. **(E)** *BCCE4*[G] cells incubated with anti-PD-L1 antibody were then cocultured with activated PBMCs at the indicated E/T ratios for 24 h, and the cytotoxicity was measured by LDH assays. E/T, effector: target. Statistical significance was assessed using two-tailed Student's *t* test. The values represent the mean ± SD. $^{*}P < 0.05$.

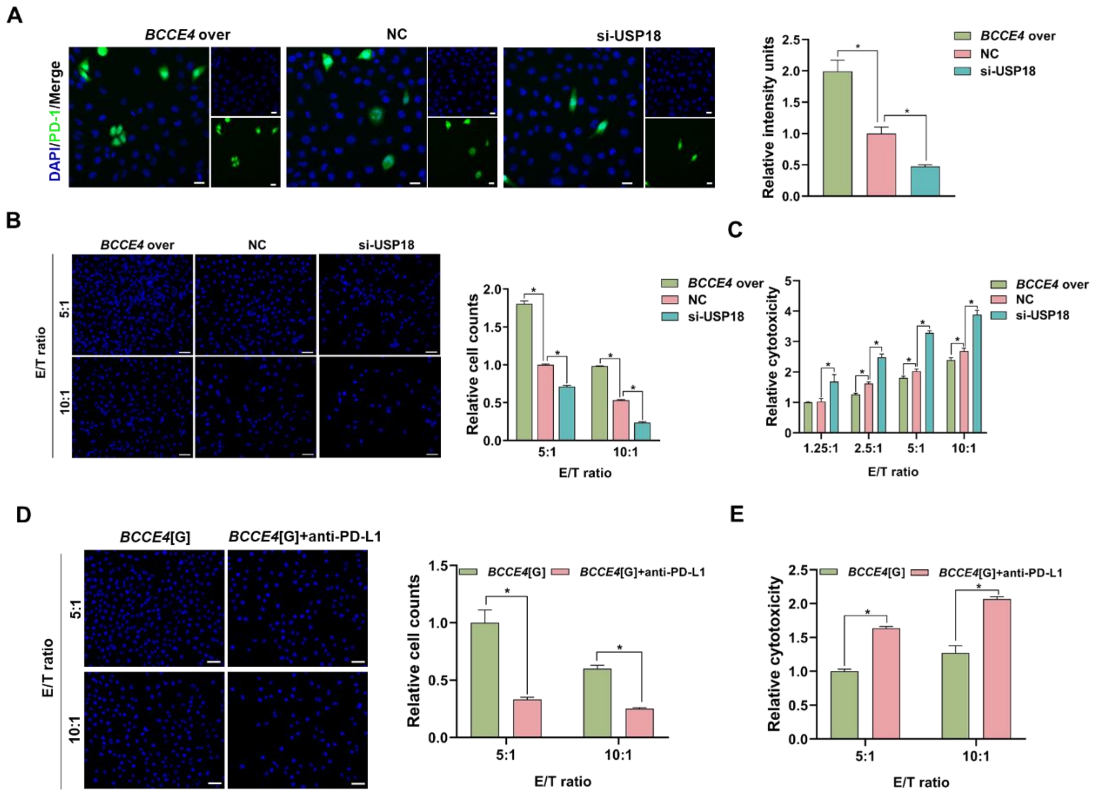
A
BCCE4 over
NC
si-USP18
DAPI/PD-1/Merge
Relative intensity units
B
E/T ratio
5:1
10:1
Relative cell counts
C
Relative cytotoxicity
1.25:1
2.5:1
D
BCCE4[G]
BCCE4[G]+anti-PD-L1
E

**Figure S31. The expression of lncRNA *BCCE4*, USP18 and PD-L1 in vivo. (A)** Harvested tumor tissues in the NC, *BCCE4*[G] and *BCCE4*[A] groups. **(B)** The protein levels of USP18 and PD-L1 in tumor tissues from NC, *BCCE4*[G] and *BCCE4*[A] groups as measured by Western blotting. **(C)** Expression of exosomal lncRNA *BCCE4* in tumor tissues from the NC, *BCCE4*[G] and *BCCE4*[A] groups. Representative images of Ki67 **(D)** and PD-L1 **(E)** immunohistochemistry results. Scale bar, 50 μm. Quantification of Ki67 **(F)**, USP18 **(G)** and PD-L1 **(H)** in tumor tissues from NC, *BCCE4*[G] and *BCCE4*[A] groups. Statistical significance was assessed using two-tailed Student's *t* test. Values represent the means ± SD. **P* < 0.05.

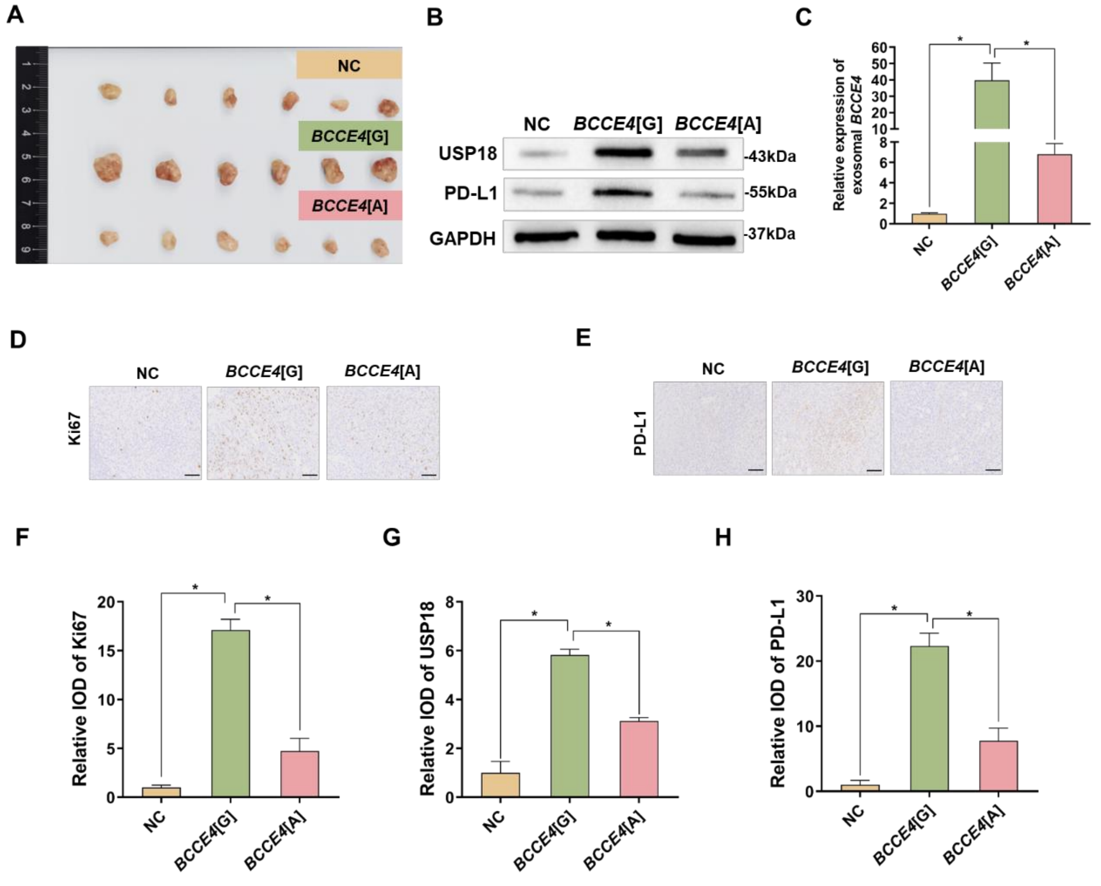

Supplement: Supplementary file 1 — Supporting Information [file ADVS-10-2303473-s001.pdf]
